# Motivational learning biases are differentially modulated by genetic determinants of striatal and prefrontal dopamine function

**DOI:** 10.1007/s00702-021-02382-4

**Published:** 2021-07-24

**Authors:** Anni Richter, Lieke de Boer, Marc Guitart-Masip, Gusalija Behnisch, Constanze I. Seidenbecher, Björn H. Schott

**Affiliations:** 1 Department of Behavioral Neurology, Leibniz Institute for Neurobiology, Brenneckestr. 6, 39118 Magdeburg, Germany; 2 Ageing Research Centre, Karolinska Institute, Stockholm, Sweden; 3 Present Address: Max Planck Institute for Human Development, Center for Lifespan Psychology, Berlin, Germany; 4 Max Planck UCL Centre for Computational Psychiatry and Ageing Research, University College London, London, UK; 5 Center for Behavioral Brain Sciences, Magdeburg, Germany; 6 Department of Psychiatry and Psychotherapy, University Medicine Göttingen, Göttingen, Germany; 7 Department of Neurology, University of Magdeburg, Magdeburg, Germany; 8 German Center for Neurodegenerative Diseases (DZNE), Göttingen, Germany

**Keywords:** Dopamine D2 receptor, TaqIA, COMT, Reward learning, Motivated learning, Action bias

## Abstract

Dopaminergic neurotransmission plays a pivotal role in appetitively motivated behavior in mammals, including humans. Notably, action and valence are not independent in motivated tasks, and it is particularly difficult for humans to learn the inhibition of an action to obtain a reward. We have previously observed that the carriers of the DRD2/ANKK1 TaqIA A1 allele, that has been associated with reduced striatal dopamine D2 receptor expression, showed a diminished learning performance when required to learn response inhibition to obtain rewards, a finding that was replicated in two independent cohorts. With our present study, we followed two aims: first, we aimed to replicate our finding on the DRD2/ANKK1 TaqIA polymorphism in a third independent cohort (*N* = 99) and to investigate the nature of the genetic effects more closely using trial-by-trial behavioral analysis and computational modeling in the combined dataset (*N* = 281). Second, we aimed to assess a potentially modulatory role of prefrontal dopamine availability, using the widely studied COMT Val108/158Met polymorphism as a proxy. We first report a replication of the above mentioned finding. Interestingly, after combining all three cohorts, exploratory analyses regarding the COMT Val108/158Met polymorphism suggest that homozygotes for the Met allele, which has been linked to higher prefrontal dopaminergic tone, show a lower learning bias. Our results corroborate the importance of genetic variability of the dopaminergic system in individual learning differences of action–valence interaction and, furthermore, suggest that motivational learning biases are differentially modulated by genetic determinants of striatal and prefrontal dopamine function.

**Supplementary Information:**

The online version contains supplementary material available at 10.1007/s00702-021-02382-4.

## Introduction

The impact of motivation on cognitive functions has been subject to intense investigation over the past 2 decades. While the influence of motivational salience on cognitive processes and goal-directed behavior is common knowledge nowadays, theories of instrumental learning have until recently neglected the influence of outcome valence on action initiation. Two logically assumed independent axes of behavioral control, namely a valence axis running from reward to punishment, and an action axis running from vigor to inhibition, have been shown to interact (Guitart-Masip et al. 2012). To study this phenomenon, a *go/no-go* task was developed that independently dissociates, i.e. orthogonalizes, action and valence, which includes the four conditions: *go to win*, *go to avoid losing*, *no-go to win,* and *no-go to avoid losing*. If the two axes of behavioral control, action and valence, would be independent, all conditions should be learned equally well. However, biased behavior, that is, an interaction of action and valence is observed, and the larger the bias the higher the coupling of action and valence, such that signals that predict reward are prepotently associated with behavioral activation, whereas signals that predict punishment are intrinsically coupled to behavioral inhibition. This finding has been robustly replicated in multiple studies (Guitart-Masip et al. 2012, 2014; Cavanagh et al. 2013; Chowdhury et al. 2013; Richter et al. 2014; de Berker et al. 2016; Swart et al. 2017, 2018; de Boer et al. 2019; Dorfman and Gershman 2019; Betts et al. 2020; Kuhnel et al. 2020; Perosa et al. 2020; van Nuland et al. 2020; Ereira et al. 2021). Understanding the neurocognitive mechanisms underlying this behavioral bias is thus important for developing more comprehensive theories of instrumental learning.

Numerous studies in a multitude of species, including humans, indicate the importance of dopamine (DA) in the neural manifestation of motivated behavior. According to a prevalent view in reinforcement learning and decision making, DA neurons signal reward prediction errors (Montague et al. 1996; Schultz et al. 1997; Bayer and Glimcher, 2005), in the form of phasic bursts for positive prediction errors and dips below baseline firing rate for negative prediction errors (Bayer et al. 2007), resulting in corresponding peaks and dips of DA availability in target structures, most prominently the striatum (McClure et al. 2003; O’Doherty et al. 2003, 2004; Pessiglione et al. 2006). In the striatum, increased DA release in response to an unexpected reward reinforces the direct pathway via activation of D1 receptors and thereby facilitates the future generation of *go* choices under similar circumstances, while dips in DA levels in response to an unexpected punishment reinforce the indirect pathway via reduced activation of D2 receptors, thereby facilitating the subsequent generation of *no-go* choices in comparable situations (Frank et al. 2004, 2007; Wickens et al. 2007; Hikida et al. 2010).

As the human dopaminergic system is subject to considerable genetic variability, several polymorphisms that have been associated with alterations in dopaminergic gene products (e.g., DRD2, COMT, DAT, and DARPP-32; see supplementary Figure S1) have been used to study naturally occurring differences in the dopaminergic system of healthy subjects. In line with the assumptions outlined above, we observed in a previous study (Richter et al. 2014) that the coupling of action and valence during learning was modulated by a genetic variant linked to striatal DA D2 receptor expression. We argued that A1 carriers with presumably less D2 receptors would be assumed to have less limitation of dopaminergic signaling after negative prediction errors in the indirect pathway and a shift to a more action-oriented behavioral pattern mediated by the direct pathway (see Fig. 4). In line with that framework, in a recent study, de Boer et al. (2019) found a positive correlation between the strength of the action by valence interaction and dorsal striatal D1 receptor availability measured using positron emission tomography (PET). Therefore, striatal dopaminergic effects may be sufficient to explain biased motivational learning (Swart et al. 2017; de Boer et al. 2019). On the other hand, Guitart-Masip et al. (2014) observed that levodopa administration led to a reduced coupling of action and valence that cannot be explained by striatal action of DA. The authors attributed their observation to an effect on prefrontal cortex (PFC) functioning, where DA plays a role in facilitating working memory and attentional processes (Seamans and Yang, 2004; Hitchcott et al. 2007; Haber and Knutson, 2010) that may help to overcome the biased behavior. This effect of levodopa administration was recently replicated in patients with non-tremor Parkinson's disease (van Nuland et al. 2020), and studies investigating frontal network dynamics using electroencephalography further demonstrate that prefrontal control processes (as indexed by higher mid-frontal theta power) are important to overcome biased behavior (Cavanagh et al. 2013; Swart et al. 2018). Therefore, DA may influence these learning biases in a regionally specific manner.

Numerous previous studies have investigated the influence of candidate single-nucleotide polymorphisms (SNPs) of DA on instrumental learning (Frank et al. 2007; Klein et al. 2007; Frank & Hutchison, 2009; Jocham et al. 2009; Corral-Frias et al. 2016). As the expression of several key molecules of the dopaminergic system shows a characteristic regional distribution in the brain, genetically mediated differences may also provide some information about the contributions of different brain regions to DA-dependent learning and memory processes (Schott et al. 2006; Mier et al. 2010; Corral-Frias et al. 2016). In the current study, we aimed to examine differential contributions of two dopaminergic SNPs: the DRD2/ANKK1 TaqIA SNP (rs1800497) and the COMT Val108/158Met SNP (rs4680).

In PET studies, the DRD2/ANKK1 TaqIA polymorphism has repeatedly been linked to lower striatal D2 binding availability in carriers of the less common A1 allele (for review and meta-analysis, see Gluskin and Mickey 2016; Eisenstein et al. 2016). With respect to motivated behavior, Stice et al. (2012) found stronger midbrain activation in A1 carriers compared with A2 homozygotes on reward expectancy, and Stelzel et al. (2010) reported generally increased striatal BOLD signaling in A1 carriers. In addition, relative to A2 homozygotes, A1 carriers showed poorer performance in avoiding actions associated with punishment and lower activations of PFC and striatum during processing of negative feedback (Klein et al. 2007; Frank and Hutchison, 2009; Jocham et al. 2009).

Furthermore, there is evidence of associations of the A1 allele with psychiatric disorders such as addictions—most notably alcohol dependence (for a meta-analysis, see Wang et al. 2013; for reviews, see Samochowiec et al. 2014 and Koeneke et al. 2020)—and ADHD (for a meta-analysis, see Pan et al. 2015). In addition, it was initially hypothesized that there was an advantage of the A1 allele in schizophrenia disorders in terms of lower risk (Dubertret et al. 2004) and better response to haloperidol (Schafer et al. 2001). However, while a meta-analysis (Yao et al. 2015) failed to confirm a significant association between schizophrenia and the TaqIA polymorphism, an association with another DRD2 SNP was reaffirmed, and findings from a genome-wide association study also support the relevance of DRD2 polymorphisms in schizophrenia disorders (Schizophrenia Working Group of the Psychiatric Genomics 2014).

Moreover, behavioral experiments and questionnaire studies have been able to show associations between the A1 allele and higher scores on the personality traits reward dependence, impulsivity, curiosity (novelty seeking), and extraversion (Noble et al. 1998; Eisenberg et al. 2007; Lee et al. 2007; Smillie et al. 2010).

Catechol-O-methyltransferase (COMT) plays a key role in the breakdown of DA in the PFC (Kaenmaki et al. 2010; Schott et al. 2010), whereas its role in striatal DA inactivation has been shown to be of lesser importance (Yavich et al. 2007; Korn et al. 2021). The frequent Val108/158Met SNP in the *COMT* gene (chromosome 22) leads to an amino acid exchange from valine (Val) to methionine (Met). In Met carriers, reduced enzymatic activity and increased prefrontal DA availability have been observed, presumably due to lower thermostability of the enzyme (Chen et al. 2004). This SNP has mainly been investigated with respect to PFC-dependent executive functions (for reviews, see Frank and Fossella, 2011; Klanker et al. 2013), and a meta-analysis of functional magnetic resonance imaging (fMRI) studies confirmed that Met carriers show more efficient performance in executive functions and higher neural activations during emotion processing (Mier et al. 2010). In the context of motivated behavior, the Met allele has been associated with more successful reward learning (for a meta-analysis, see Corral-Frias et al. 2016). Moreover, Met allele carriers adapt behavior more rapidly on a trial-to-trial basis during reinforcement learning (Frank et al. 2007; Frank and Hutchison 2009).

We have previously shown in two independent cohorts that carriers of the A1 allele of the DRD2/ANKK1 TaqIA polymorphism show a rather selective deficit in learning to inhibit an action to receive a reward (Richter et al. 2014). With our present study, we followed two aims: first, we aimed to replicate our finding on the TaqIA polymorphism in a third independent cohort and to investigate the nature of the genetic effects more closely using trial-by-trial behavioral analysis and computational modeling in the combined dataset (*N* = 281). Second, we aimed to assess a potentially modulatory role of prefrontal DA availability, using the widely studied COMT Val108/158Met polymorphism as a proxy. Regarding the DRD2/ANKK1 TaqIA SNP, we hypothesized that, in line with our previous observations (Richter et al. 2014), A1 carriers would show a higher coupling of action and valence. With respect to the COMT polymorphism, we hypothesized that, given the preferential role of COMT in PFC versus striatal DA availability, carriers of the low-activity Met allele would more readily overcome the learning bias and show less coupling of valence with action.

## Materials and methods

### Participants

In addition to our previously described two cohorts of 87 and 95 participants (Richter et al. 2014), 99 newly recruited participants were tested (55 females and 44 males; age: range 20–34 years, mean 25.2 years, SD = 2.6 years; demographic description of all three samples in Supplementary Table S1). According to self-report, all participants were of European ethnicity, right-handed, had obtained at least a university entrance diploma (Abitur) as educational certificate, had no present or past neurological or mental disorder, alcohol or drug abuse, did not use centrally acting medication, and had no history of psychosis or bipolar disorder in a first-degree relative. Additionally, given the design of the experiment, regularly gambling was defined as an exclusion criterion for participation.

All participants gave written informed consent in accordance with the Declaration of Helsinki and received financial compensation for participation. The study was approved by the Ethics Committee of the Faculty of Medicine at the Otto von Guericke University of Magdeburg.

### Genotyping

Genomic DNA was extracted from blood leukocytes using the KingFisher^™^ Duo Prime Purification System (Thermo Scientific^™^) according to the manufacturer’s protocol. Genotyping of the SNPs DRD2/ANKK1 TaqIA (NCBI accession number: rs1800497) and COMT Val108/158Met (rs4680) was performed using PCR-based restriction fragment length analysis according to previously described protocols (Schott et al. 2006; Wimber et al. 2011; Richter et al. 2013, 2014, 2017). A1 carriers of the TaqIA SNP were grouped together (A1 + : A1/A1 and A1/A2; A1 − : A2/A2) as in the previous studies (Klein et al. 2007; Frank and Hutchison, 2009; Jocham et al. 2009; Stelzel et al. 2010; Stice et al. 2012; Richter et al. 2013, 2014, 2017).

### Paradigm

**Fig. 1:**
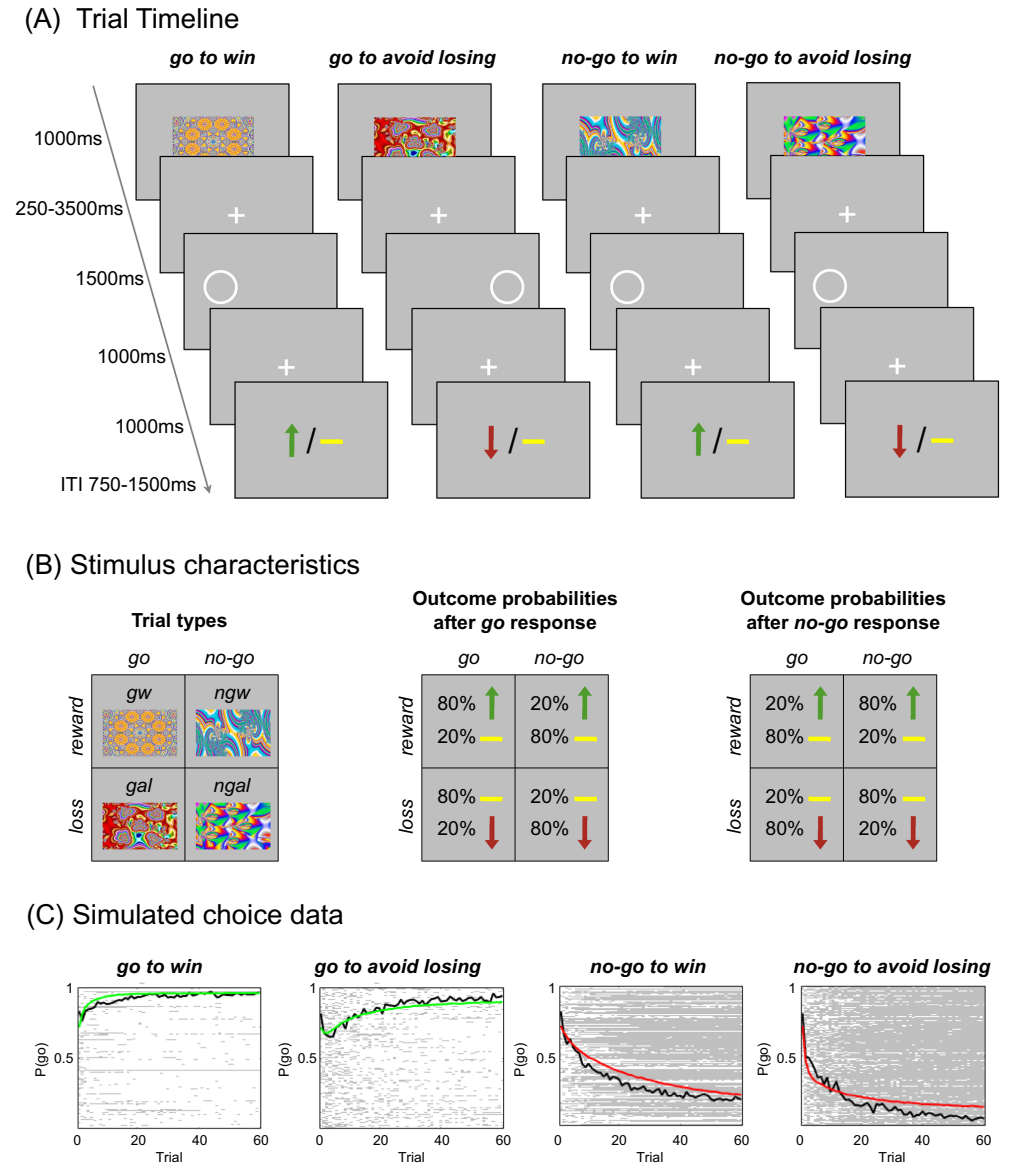
Experimental paradigm and participant performance. **A** Probabilistic monetary *go/no-go* task. Fractal cues indicate the condition—a combination of action (*go* or *no-go*) and valence (*reward* or *punishment*). On *go* trials, subjects press a button for the side of a circle. On *no-go* trials, they withhold a response. Arrows indicate *rewards* (upward) or *punishments* (downward). Horizontal bars symbolize the absence of a *reward* or *punishment*. ITI, intertrial interval. **B** The schematics represent for each condition the nomenclature (left), the possible outcomes and their probabilities after a *go* response (middle), and the possible outcomes and their probability after a *no-go* response (right). **C** Simulated choice data according to the model parameters of the winning model. Colored lines represent the simulated group mean probability of performing a *go* on each trial (green for *go* conditions, where *go* is the correct response; red for *no-go* conditions, where *no-go* is the correct response). Black lines indicate the group mean for participants’ actual *go* responses on each trial. In the plot area, each row represents one participant’s choice behavior for each trial (281 × 60 pixels). A white pixel reflects that a participant chose *go* on that trial; a gray pixel represents *no-go*. Participants made more *go* responses to *win* vs. *avoid losing* cues, reflecting the motivational bias. Overall, they successfully learned whether to make a *go* response or not (proportion of *go* responses increases for *go* cues and decreases for *no-go* cues). Figures (**A**) and (**B**) adapted from Richter et al. (2014)

We used a previously employed *go/no-go* learning task with orthogonalized action requirements and outcome valence (Guitart-Masip et al. 2012). Detailed descriptions of the task have been presented previously (Richter et al. 2014; Betts et al. 2020). Figure 1A displays the trial timeline. Briefly, each trial consisted of the presentation of a fractal cue, a target detection task, and a probabilistic outcome. First, one out of four abstract fractal cues was displayed. Prior to the beginning of the task, participants were informed that a fractal indicated i) whether they would subsequently be required to perform a target detection task by pressing a button (*go*) or not (*no-go*) and ii) the possible valence of the outcome of the subjects’ behavior (reward/no reward or punishment/no punishment). Importantly, subjects were not instructed with respect to the contingencies of each fractal image and had to learn them by trial and error. There were four trial types: press the correct button in the target detection task to gain a reward of 0.50 € [“*go to win*” (*gw*)]; press the correct button to avoid a punishment of − 0.50 € [“*go to avoid losing*” (*gal*)]; do not press a button to gain a reward [“*no-go to win*” (*ngw*)]; do not press a button to avoid punishment [“*no-go to avoid losing*” (*ngal*)]. The outcome was probabilistic (see Fig. 1B). To avoid incidental effects of specific cue images, the association of the fractal images with the specific conditions (go vs. no-go* reward vs. punishment) was randomized across participants. The task included 240 trials (60 trials per condition) and was divided into four sessions. Subjects were told that they would be paid their earnings of the task up to a total of 25 € and a minimum of 7 €. Before starting the actual learning task, subjects performed 10 trials of the target detection task to familiarize themselves with the speed requirements.

### Statistical analysis

Accuracy was analyzed using IBM^®^ SPSS^®^ Statistics version 21. The percentage of correct choices in the target detection task (button press in *go* trials and omission of responses in *no-go* trials) was collapsed across time bins of 30 trials per condition. To assess the learning enhancement, the slope was calculated by substracting the mean values in the first half of the experiment from the mean values of the second half of the experiment $\left( {{\text{slope}}\; = \;{\text{mean}}\;\left[ {{\text{2nd}}\;{\text{half}}} \right]\; - \;{\text{mean}}\left[ {{\text{1st}}\;{\text{half}}} \right]} \right)$.

For the replication of our previous study (Richter et al. 2014) in the new cohort (*N *= 99), we compared DRD2/ANKK1 TaqIA genotype groups with a *t* test for independent samples and investigated task effects with a mixed analysis of variance (ANOVA) with time (1st/2nd half), action (*go/no-go*), and valence (*win*/*avoid losing*) as within-subject factors.

Then, by combining all three datasets (*N *= 281), we included the two genotypes as between-subject factors in the analysis and added cohort (three cohorts represented in two dichotomous dummy coded variables for cohort 2 and 3), and age and gender as covariates (analysis of covariance, ANCOVA). The increased number of participants allowed us to run a logistic regression on the trial-by-trial *go* responses as in Swart et al. (2017) which more accurately analyzes the data, as it is closer to the actual behavior of each participant by including inter- and intraindividual variability (see supplementary methods for details).

Unless stated otherwise, independent samples *t* tests were used as post hoc tests, and the significance threshold was set to 0.05, two-tailed. Whenever Levene’s test was significant, statistics were adjusted, but for better readability, uncorrected degrees of freedom are reported.

### Computational modeling of task performance

Computational modeling of task performance was employed using MATLAB^®^ R2016B (Mathworks^®^). We used a previously published modeling procedure (Huys et al. 2011; Guitart-Masip et al. 2012). Detailed descriptions of the reinforcement learning models as well as the model fitting procedure and comparison have been described in a recent study of age effects in the same task (Betts et al. 2020). Briefly, we constructed six nested reinforcement learning models to fit participants’ behavior (Table 2). The base model was a Q-learning algorithm (Sutton and Barto 1998) that used a Rescorla–Wagner update rule to independently track the action value of each choice (*go; no go*), given each fractal image, with a learning rate (*ε*) as a free parameter. In this model, the probability of choosing one action on a trial was a sigmoid function of the difference between the action values scaled by a slope parameter that was parameterized as sensitivity to reward (*ρ)*. This basic model was augmented with an irreducible noise parameter (*ξ*) and then further expanded by adding a static bias parameter to the value of the *go* action (*b*). Furthermore, we allowed for separate sensitivities to rewards (*ρ*_*win*_) and punishments (*ρ*_*lose*_). As in our recent study of age effects (Betts et al. 2020), the model was then extended by adding a constant Pavlovian value of 1 or − 1 to the value of the *go* action as soon as the first reward for *win* cues or the first punishment for *avoid losing* cues, respectively, was encountered. This fixed Pavlovian value was weighted by a further free parameter (Pavlovian parameter) into the value of the *go* action (*π*). Model comparisons demonstrated a better fit compared to a variable Pavlovian value used in the previous studies (Guitart-Masip et al. 2012; Cavanagh et al. 2013; de Boer et al. 2019) (see Table 2). As in the previous reports (Huys et al. 2011; Guitart-Masip et al. 2012), we employed a hierarchical Type II Bayesian procedure using maximum likelihood to fit simple parameterized distributions for higher level statistics of the parameters. All six computational models were fit to the data using a single distribution for all participants. This fitting procedure was, therefore, blind to the existence of different genotype groups with putatively different parameter values. Models were compared using the integrated Bayesian Information Criterion (iBIC) with small iBIC values indicating a model that fits the data better after penalizing for the number of data points associated with each parameter. Finally, we assessed genotype-related effects on all modeling parameters using IBM^®^ SPSS^®^ Statistices version 21. To test for differences regarding specific model parameters, we calculated *t* tests for independent samples. As one could not exclude that not one specific parameter but a combination of them differed between genotypes, we performed a multivariate test of differences—a linear discriminant analysis (LDA). The purpose of LDA was to find a linear combination of the six model parameters that gives the best possible separation between the genotype groups. This method simultaneously accounts for differences in combinations of variables between groups over and beyond differences across single multiple variables (Ramos and Liow 2012).

## Results

### Reduced learning performance in DRD2/ANKK1 TaqIA A1 carriers

**Fig. 2:**
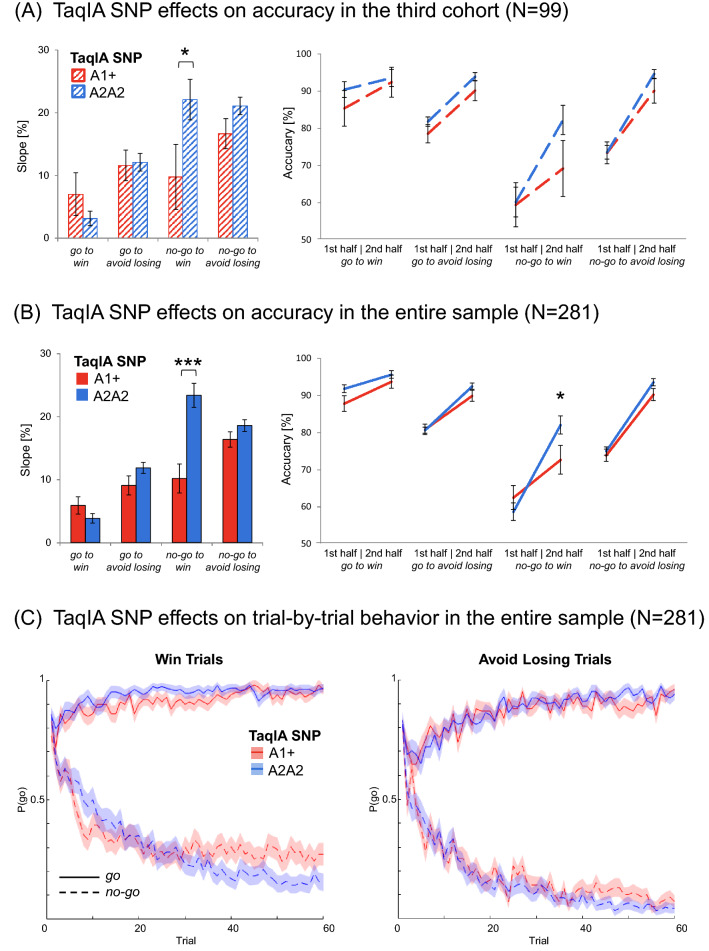
Effects of DRD2/ANKK1 TaqIA genotype on choice performance. **A** and **B** Effects of DRD2/ANKK1 TaqIA genotype on choice performance in the third cohort (*N *= 99) and in the entire sample (*N *= 281). Compared to the A2 homozygotes, A1 carriers showed a diminished learning to withhold an action to receive a reward. Left panels: bar plots show mean differences between correct response rates (± SEM) during second half versus the first half of trials for each condition. This score represents the observed fourfold interaction of *action* × *valence* × *time* × *genotype*. Right panels: line charts show mean values of correct responses (± SEM) in the first and the second half of trials for all four conditions. Post hoc comparisons via *t* tests: *p < 0.05, ***p < 0.001. **C** Trial-by-trial proportions of *go* responses (± SEM) to *go* cues (solid lines) and *no-go* cues (dashed lines) across cue types. *Win* and *avoid losing* condition seperately and colors depict DRD2/ANKK1 TaqIA genotypes. TaqIA A1 carriers showed an enhanced effect of cue valence on *go* responding especially in the *no-go to win* condition with further progress of the experiment (lines are mostly separated). Adapted scripts of Swart et al. (2017) were used to generate figures

In our previous study (Richter et al. 2014), we observed that in the *no-go to win* condition, DRD2/ANKK1 TaqIA A1 carriers showed a significantly diminished improvement from the first to the second half of the experiment compared to A2 homozygotes (cohort 1: *t*_85_ =  − 2.78, *p* = 0.007; cohort 2: *t*_93_ =  − 2.16, *p* = 0.033). As expected, we replicated this finding in our current sample (cohort 3: *t*_97_ = 2.05, *p* = 0.043; Fig. 2A). In all other conditions, A1 carriers and A2 homozygotes did not significantly differ (all *p* > 0.100), nor in gender (*p* = 0.621), age (*p* = 0.749), the number of smokers and nonsmokers (*p* = 0.084), or in the COMT Val108/158Met genotype distribution (*p* = 0.901).

Furthermore, we also analyzed task effects and replicated previous results showing an action by valence interaction on overall task performance (Guitart-Masip et al. 2012, 2014; Cavanagh et al. 2013; Chowdhury et al. 2013; Richter et al. 2014; de Berker et al. 2016; Swart et al. 2017, 2018; de Boer et al. 2019; Dorfman and Gershman, 2019; Betts et al. 2020; Kuhnel et al. 2020; Perosa et al. 2020; van Nuland et al. 2020; Ereira et al. 2021); see supplementary results and Table S2 for details).

### Genotyping results in the entire sample

Our further analyses of genetically driven effects were performed in the entire sample comprising all three cohorts (*N *= 281 participants). Within this group, 99 carriers of the DRD2/ANKK1 A1 allele (35.2%; 10 A1/A1 and 89 A1/A2) and 182 A2 homozygotes were identified. For the COMT Val108/158Met polymorphism, 83 subjects were Met homozygous, 70 subjects were Val homozygous, and the remaining 128 subjects were heterozygous. These distributions are within the expected range for a European population (see Supplementary Table S3; NCBI ALFA project release version: 20201027095038; (Phan et al. 2020). Genotype frequencies were in Hardy–Weinberg equilibrium (all *p* > 0.145), and there was no linkage between the two polymorphisms (*p* = 0.971; for detailed demographics, see Table 1).

**Table 1:** Descriptive data of the entire sample regarding DRD2/ANKK1 TaqIA and COMT Val108/158Met genotypes

DRD2/ANKK1 TaqIA	A1 +	A1 −	A1 + > A1 −
Gender (*N* women/men)	44/55	102/80	*χ*^*2*^ = 3.46 *p* = .063
Age in years (M ± SD)	25.1 ± 3.1	24.6 ± 2.6	*t*_*279*_ = 1.30 *p* = .195
Non-smokers/smokers (*N*)	70/29	143/39	*χ*^*2*^ = 2.16 *p* = .141
COMT (*N* MM/VM/VV)	30/45/24	53/83/46	*χ*^*2*^ = 0.06 *p* = .971

COMT Val108/158Met	MM	VM	VV	MM > VV	VM > VV	MM > VM
Gender (*N* women/men)	43/40	64/64	39/31	*χ*^*2*^ = 0.59, *p* = .743		
Age in years (M ± SD)	25.0 ± 2.7	24.9 ± 2.8	24.3 ± 3.0	*t*_151_ = 1.36 *p* = .176	*t*_196_ = 1.37 *p* = .174	*t*_209_ = 0.09 *p* = .931
Non-smokers/smokers (*N*)	63/20	93/35	57/13	*χ*^*2*^ = 1.90, *p* = .387		
TaqIA (*N* A1 + /A1 − )	30/53	45/83	24/46	*χ*^*2*^ = 0.06, *p* = .971		

Demographic data are pooled across all three cohorts (cohort 1 and 2 from Richter et al. (2014), and the newly investigated cohort 3). *N *= number, M = mean, SD = standard deviation, VM: Val/Met heterozygotes, VV: Val homozygotes, A1 + : carriers of the A1 allele, A1 − : A2 homozygotes

To further control for effects of population stratification, genotyping was also performed for a variety of additional polymorphisms with a known distribution in European populations (see Supplementary Table S3). The distributions were in line with previously reported frequencies and did not differ between genotype groups of the DRD2/ANKK1 and COMT polymorphisms (all *p* > 0.112), thus making genetic inhomogeneity of the tested population unlikely.

### DRD2/ANKK1 TaqIA and COMT genotypes differentially modulate motivational learning biases

In line with our previous work (Richter et al. 2014), we observed for the DRD2/ANKK1 TaqIA SNP a significant *genotype* × *time* × *action* × *valence* interaction (*F*_1,271_ = 11.18, *p* = 0.001; see Fig. 2B), as well as significant interactions of *genotype* × *time* (*F*_1,271_ = 11.08, *p* = 0.001) and *genotype* × *time* × *action* (*F*_1,271_ = 11.94, *p* = 0.001). *Post hoc* comparisons revealed that A1 carriers exhibited an overall significantly worse learning performance throughout the experiment compared to A2 homozygotes (overall slope: *t*_279_ =  − 3.72, *p* < 0.001, Cohen’s *d* = 0.47). This effect was solely carried by the *no-go* conditions (*no-go* slope: *t*_279_ =  − 4.56, *p* < 0.001, Cohen’s *d* = 0.58; *go* slope: *p* = 0.748), and specifically by the *no-go to win* condition (*ngw* slope: *t*_*279*_ =  − 4.41, *p* < 0.001, Cohen’s *d* = 0.54; all other conditions: all *p* > 0.087). As displayed in Fig. 2B and C, the DRD2/ANKK1 TaqIA A1 carriers reached their learning asymptote earlier and to a lower level. They significantly differed in performance from the A2 homozygotes only during the second half of the experiment, pointing to different learning capacities (overall 2nd half:* t*_279_ =  − 2.21, *p* = 0.028, Cohen’s *d* = 0.35; *no-go* 2nd half: *t*_279_ =  − 2.28, *p* = 0.024, Cohen's *d* = 0.29; *ngw* 2nd half: *t*279 =  − 2.06, *p* = 0.041, Cohen’s *d* = 0.26; equivalent 1st half comparisons: all *p* > 0.340). A summary of the statistics is displayed in Supplementary Tables S4 and S5.

The combined datasets allowed for a logistic regression on the trial-by-trial *go* responses (see supplementary results and Figure S2 for details). This analysis confirmed the ANCOVA results with A1 carriers showing significantly diminished *no-go to win* performance in the course of the experiment (Fig. 2C).

**Fig. 3:**
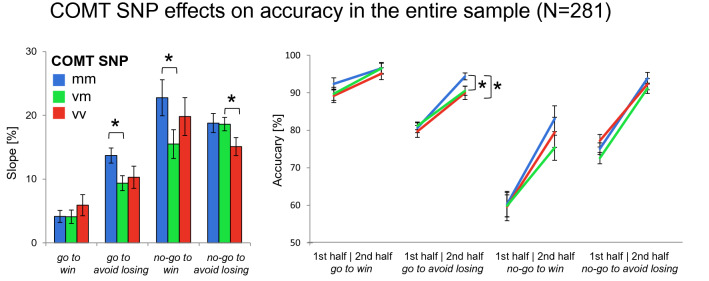
Effects of COMT genotype on choice performance in the entire sample. Left panels: bar plots show mean differences between correct response rates (± SEM) during second half versus the first half of trials for each condition. This score represents the observed fourfold interaction of *action* × *valence* × *time* × *genotype*. Right panels: line charts show mean values of correct responses (± SEM) in the first and the second half of trials for all four conditions. Met homozygotes showed increased learning throughout the experiment in the *no-go to win* and *go avoid losing* condition relative to heterozygotes. Post hoc comparisons via *t* tests: *p < 0.05

For the COMT Val108/158Met polymorphism, we observed a trend toward a significant four-way interaction *genotype* × *time* × *action* × *valence* (*F*_2,271_ = 2.96, *p* = 0.053). Met homozygotes showed significantly increased learning throughout the experiment in the *no-go to win* (*ngw* slope: *t*_209_ = 2.02, *p* = 0.045; Fig. 3) and the *go to avoid losing* conditions (*gl* slope: *t*_209_ = 2.48, *p* = 0.014) compared to heterozygotes (other conditions: all *p* > 0.922). The logistic regression did not show an effect of COMT genotype (*p* = 0.381; see supplementary results and Figure S3 for details).

In light of previous evidence that Met homozygotes have a higher response bias relative to Val carriers (Lancaster et al. 2012, 2015; Goetz et al. 2013; Corral-Frias et al. 2016), in an additional analysis, participants were separated into Met homozygotes (Met/Met) and Val allele carriers (Val/Val and Val/Met). The ANCOVA revealed a significant *genotype* × *time* × *action* × *valence* interaction (*F*_1,273_ = 4.30, *p* = 0.039) as well as a significant main effect of COMT genotype (*F*_1,273_ = 4.55, *p* = 0.034) and interestingly also a significant interaction of the COMT with the TaqIA genotype (*F*_1,273_ = 3.88, *p* = 0.050). The latter finding indicates a beneficial effect of Met homozygosity on overall performance in A1 carriers (*t*_97_ = 2.31, *p* = 0.024) but not in A2 homozygotes (*p* = 0.971).

We controlled for potential effects in reaction times (participants were explicitly instructed to respond accurately) and false responses in the target detection task (i.e., left when the target was on the right side of the display or vice versa) and found no significant differences between genotype groups (*p* > 0.187; see supplement for details).

### Computational modeling of task performance

To identify components of the observed asymmetry during learning, we constructed six nested reinforcement learning models to fit participants’ behavior (Table 2). Our computational modeling approach demonstrated that the marked asymmetry in learning could be best accounted for by the model including separate parameters for sensitivity to rewards and punishments as well as a learning rate, an irreducible noise parameter, a constant *go* bias parameter, and a constant Pavlovian bias parameter (see Table 2), which is consistent with our recently published lifetime study on motivational learning (Betts et al. 2020). The simulations of the winning model are presented in Fig. 1C. Neither one specific model parameter (independent samples *t* tests: all* p* > 0.119), nor a linear combination of the parameters (LDA: all *p* > 0.636) showed significant genotype-related differences.

**Table 2:** Integrated Bayesian information criteria (iBIC) for tested models

Model no	Model parameters	No. of parameters	Likelihood	Pseudo-*R*^2^	iBIC
1	*ε, ρ*	2	− 23,463	0.498	46,970
2	*ε, ρ, ξ*	3	− 23,314	0.501	46,695
3	*ε, ρ, ξ, b*	4	− 21,798	0.534	43,685
4	*ε, ρ*_*win*_*, ρ*_*lose*_*, ξ, b*	5	− 21,334	0.544	42,779
5	*ε, ρ*_*win*_*, ρ*_*lose*_*, ξ, b, π*_*variable*_	6	− 21,137	0.548	42,406
**6**	***ε, ρ***_***win***_***, ρ***_***lose***_***, ξ, b, π***_***constant***_	**6**	− **21,106**	**0.549**	**42,346**

Boldface type: winning model statistics, *ε*: learning rate, *ρ*_*win*_: weighting of reward on win trials, *ρ*_*lose*_: weighting of punishments on lose trials. *ξ*: irreducible noise, b: go bias, *π*: Pavlovian bias, iBIC: integrated Bayesian information criterion (smaller iBIC values indicate a better model fit). Descriptives for the parameters in the winning model (M ± SD): *ε* = 0.26 ± 0.15, *ρ*_*win*_ = 15.32 ± 13.30, *ρ*_*lose*_ = 7.51 ± 4.03, ξ = 0.96 ± 0.06, b = 1.10 ± 0.74, *π*_*constant*_ = 0.65 ± 0.57

## Discussion

In the present study, we investigated how genetic determinants of striatal and prefrontal DA function modulate learning biases when action and valence are experimentally orthogonalized. Using the previously established valenced *go/no-go* task (Guitart-Masip et al. 2012), we provide independent confirmation for a selective deficit of DRD2/ANKK1 TaqIA A1 carriers in learning to inhibit an action to obtain a reward. Moreover, our exploratory analysis yielded preliminary evidence that COMT Met homozygotes show superior learning during trials with incongruent coupling of action and valence. Due to previous knowledge about their neurophysiological consequences, the genetic polymorphisms studied here allow conclusions about differential contributions of striatal and prefrontal DA function to instrumental control mechanisms (Schott et al. 2006; Mier et al. 2010; Corral-Frias et al. 2016).

### Selective modulation of the *no-go to win* condition by DRD2/ANKK1 TaqIA genotype

For the DRD2/ANKK1 TaqIA polymorphism, we replicated our previous observation (Richter et al. 2014) that A1 carriers show a stronger coupling of action and valence in a third independent cohort. As in our previous study, A1 carriers exhibited a specific impairment in learning to withhold actions in reward contexts. When combining all three datasets (*N *= 281), we could more closely investigate the nature of this effect.

D2-type DA receptors are primarily expressed in the striatum (*post-mortem* autoradiography: Joyce et al. 1991; Kessler et al. 1993; Hall et al. 1996; in vivo PET: Okubo et al. 1999; MacDonald et al. 2009). They function as both postsynaptic inhibitory receptors and as presynaptic autoreceptors that regulate neurotransmission via negative feedback (Bello et al. 2011, for reviews, see Wolf and Roth, 1990; Schmitz et al. 2003). While DRD2 is, albeit sparsely, expressed in extrastriatal regions (2–8% of the expression level in the striatum, Suhara et al. 1999) and cortically mediated effects can thus not be excluded, differences for the DRD2/ANKK1 TaqIA genotypes have thus far only been observed for the striatum—with lower DRD2 expression or binding availability in A1 carriers (*post-mortem* autoradiography: Noble et al. 1991; Thompson et al. 1997; Ritchie and Noble, 2003; in vivo PET: for review and meta-analyis, see Gluskin and Mickey 2016; Eisenstein et al. 2016).

**Fig. 4:**
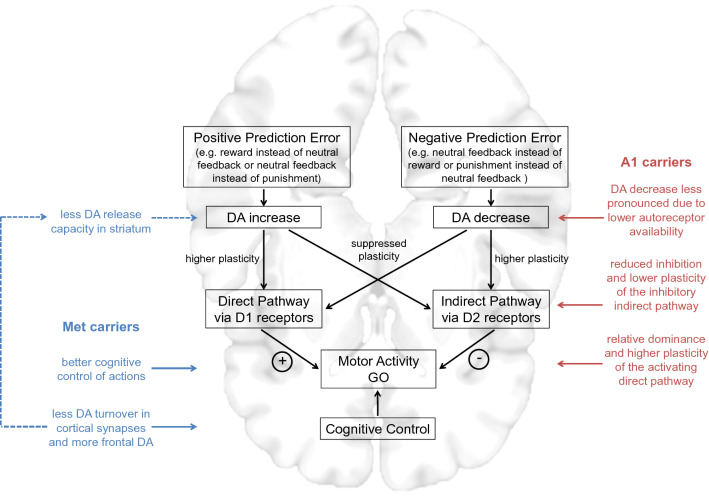
A model of genetically driven contributions to the coupling of action and valence during learning. DA neurons signal positive reward prediction errors by phasic bursts and negative prediction errors by dips below baseline firing rate. While the first reinforces the direct pathway via activation of D1 receptors and thereby facilitates the future generation of *go* choices, the second reinforces the indirect pathway via reduced activation of D2 receptors and thus facilitates the future generation of *no-go* choices in comparable situations. A1 carriers would be assumed to have reduced D2 receptor-binding capacity decreasing autoinhibition of dopaminergic signaling after negative prediction errors in the indirect pathway and a shift to a more action-oriented behavioral pattern mediated by the direct pathway. COMT Val108/158Met Met carriers would be assumed to have higher frontal DA availability facilitating working memory and attentional processes. Moreover, indirect downstream effects on striatal DA regulation may add on improving performance under Pavlovian conflict in Met compared to Val homozygotes. The MNI template brain from MRIcroGL (“mni152”) was used in this illustration. Figure adapted from Richter et al. (2014)

Those techniques cannot differentiate between presynaptic and postsynaptic D2 receptors. Thus genetically mediated differences in dopamine-dependent learning processes may to some extent be attributable to reduced availability of presynaptic autoinhibitory D2 receptors, which in turn may underlie the previously reported increased DA synthesis capacity in A1 carriers (Laakso et al. 2005; Fig. 4). Two SNPs of the DRD2 gene, rs2283265 and rs1076560, have previously been associated with alternative splicing and a rather selective decrease of presynaptic D2 receptor expression (Zhang et al. 2007). Notably, in a motivational learning study, the haplotype linked to lower presynaptic D2 receptor availability was associated with relatively impaired avoidance learning, but intact approach learning (Frank and Hutchison 2009). However, it is not possible to separate in this study whether the effects were actually due to the aversive nature of the feedback or to poorer *no-go* learning, because there was no control of the coupling of action and valence. Nevertheless, that finding is compatible with the possibility that the rather selective deficit of A1 carriers in the *no-go to win* condition observed in the present study may, at least in part, be attributable to reduced presynaptic D2 receptor density.

Another factor that comes into play are the assumed different functions in reward learning of dorsal striatal regions that include the caudate nucleus and putamen specifically involved in learning about actions and their reward consequences, and ventral striatal regions, encompassing the nucleus accumbens classically linked to expected value representations (Wickens et al. 2003, 2007; O'Doherty et al. 2004).

While differences in DRD2 binding availability of DRD2/ANKK1 TaqIA A1 allele carriers have been observed for all striatal subregions (putamen, caudate, and nucleus accumbens; Eisenstein et al. 2016), studies using the valenced *go/no-go* learning task investigating regionally specific striatal functions thus far only observed correlations with the dorsal striatum. De Boer et al. (2019) investigated cortical and striatal sources of variance in D1 receptor availability in humans using PET and could show that higher levels of endogenous D1 receptor availability in the dorsal striatum were related to biases during learning. Perosa et al. (2020) analyzed voxel-based morphometry using 7 Tesla MRI images and could show that individual differences in learning rate in older adults were related to the volume of the caudate nucleus. Relatedly, an fMRI study in young adults using a variation of the task that does not require learning (Guitart-Masip et al. 2011) demonstrated an association between the anticipation of action value and activity in the dorsal striatum suggesting its crucial role for evaluating the weight of an action. Thus, it is tempting to speculate that the observed effects of the DRD2/ANKK1 TaqIA genotype on motivational biases may be more related to dorsal striatal action learning as compared to ventral striatal functions in reward value representations, but clearly future studies are needed to answer this issue.

### Effects of the COMT Val108/158Met polymorphism and a potential role for prefrontal dopamine

Beyond replicating and expanding our findings on the DRD2/ANKK1 TaqIa polymorphism, the larger sample size of our three combined samples made it possible to investigate the effects of and potential interactions with the COMT Val108/158Met polymorphism.

The role of COMT in DA clearance has been subject to extensive research since the first studies suggesting a role for the COMT Val108/158Met polymorphism in human PFC function (Egan et al. 2001; Weinberger et al. 2001). Despite some evidence for a role for membrane-bound COMT in striatal DA metabolism (Laatikainen et al. 2013), converging evidence from animal studies and human *post-mortem* investigations suggests that COMT is primarily important for DA inactivation in the PFC, whereas its role in the striatum appears to be quantitatively negligible in most cases (Huotari et al. 2002; Matsumoto et al. 2003; Yavich et al. 2007; Kaenmaki et al. 2010; Korn et al. 2021). This has been attributed to the sparse cortical expression of the DA transporter (DAT; Chen et al. 2004; Kaenmaki et al. 2010; Tunbridge, 2010). Therefore, the COMT polymorphism has mostly been studied in relation to PFC-dependent executive functions (for reviews, see Frank and Fossella 2011; Klanker et al. 2013; for a meta-analysis, see Mier et al. 2010). With respect to motivated behavior, homozygosity for the Met allele has been associated with relatively increased reward learning (for a meta-analysis, see Corral-Frias et al. 2016). In our study, Met homozygosity is associated with stronger learning enhancement during Pavlovian conflict (i.e., incongruent coupling of action and valence) throughout the experiment—thus, improved performance when motivational biases are involved. This may be related to COMTs impact on prefrontal DA levels and prefrontal function. It should be noted, though, that despite the majority of studies showing a minor role for COMT in striatal DA metabolism, there is evidence for a delicately balanced mutual regulation of prefrontal and striatal DA turnover (Akil et al. 2003). Animal studies suggest that transgenic mice with increased COMT activity, equivalent to the relative increase in activity observed with the human COMT Val allele, do not only show deficits in PFC-dependent tasks (e.g., stimulus–response learning and working memory), but also increased DA release capacity in the striatum (Simpson et al. 2014). This finding corroborates earlier human neuroimaging studies that reported higher midbrain DA synthesis capacity in Val compared to Met homozygotes (Akil et al. 2003; Meyer-Lindenberg et al. 2005). Therefore, to the extent that the COMT genotype affects prefrontal function, it may contribute to motivational learning not only because of its biological effects in the PFC but also because of indirect downstream effects on striatal DA regulation (Fig. 4). Thus, compared with the Val allele, the Met allele, which is likely associated with relatively increased prefrontal DA signaling, would result in relatively decreased disinhibition of mesencephalic DA activity, e.g., in neuronal populations projecting to the striatum (Akil et al. 2003; Fig. 4).

### Limitations

A limitation in the interpretation of our data that is also common in other studies on this topic lies in the fact that the molecular mechanisms underlying the observed effects are still under debate. It is well known that the TaqIA polymorphism is not located within the DRD2 gene but 10 kb downstream of its termination codon on chromosome 11q23.1, within the coding region of the adjacent ankyrin repeat and kinase domain containing 1 (*ANKK1*) gene (Dubertret et al. 2004; Neville et al. 2004). The molecular mechanisms underlying the effects of ANKK1 TaqIA on striatal DRD2 availability have not been conclusively established. Multiple mechanisms have been proposed, including linkage disequilibrium (Duan et al. 2003; Ritchie and Noble, 2003; Fossella et al. 2006; Doehring et al. 2009; Richter et al. 2017) or a potential direct interaction of ANKK1 with the D2 receptor at protein level, potentially modulated by the TaqIA polymorphism (Hoenicka et al. 2010; Garrido et al. 2011; Ponce et al. 2016); for a review, see Ponce et al. 2009; see Supplementary Discussion for details). Similarly, for the COMT Val108/158Met polymorphism, it remains to be determined how COMT-dependent DA inactivation in brain regions with low DAT expression is realized. There is only limited evidence for extracellular activity of membrane-bound COMT (Chen et al. 2011), and the predominant evidence points to intracellular orientation and activity, requiring a DAT-independent uptake mechanism (Myohanen et al. 2010; Schott et al. 2010; see Supplementary Discussion).

Moreover, we only investigated two dopaminergic SNPs, and it must be noted that there are several additional genetic variants in the dopaminergic system that could be important for the generation and overcoming of motivational learning biases. In the Supplementary Discussion, we summarize the previous results on motivated behavior, focusing on the commonly investigated DAT1 VNTR rs28363170, the DARPP-32 rs907094, and the DRD2 C957T rs6277 polymorphism. Owing to the sample size, those polymorphisms were not investigated in the present study.

A further limitation lies in our modeling approach, which failed to reflect the very robust and replicated effect of the DRD2/ANKK1 TaqIA SNP on learning gain throughout the experiment in the *no-go to win* condition and on the time-dependent valence effect on individual *go/no-go* responses. One explanation could be that the model space does not include the computational mechanism to differentiate, for example, instrumental from Pavlovian contributions. This should be addressed in future studies.

### Conclusion

With our study, we demonstrate by assessing the contributions of two well-studied genetic polymorphisms that DRD2/ANKK1 TaqIA A1 carriers with presumably reduced striatal D2 receptor-binding capacity and less autoinhibition of striatal dopaminergic signaling after negative prediction errors in the indirect pathway showed a shift to a more action-oriented and biased behavioral pattern. COMT Val108/158Met Met homozygotes, who presumably exhibit higher prefrontal DA activity, showed less biased learning, possibly reflecting more efficient frontal control.

## Supplementary Information

Below is the link to the electronic supplementary material.

Supplementary file1 (DOCX 902 KB)

## Declarations

### Data and code availability

The datasets generated during and/or analyzed during the current study as well as the code are available from the corresponding author on reasonable request.
