# Correction to: Motivational learning biases are differentially modulated by genetic determinants of striatal and prefrontal dopamine function

**DOI:** 10.1007/s00702-021-02398-w

**Published:** 2021-08-20

**Authors:** Anni Richter, Lieke de Boer, Marc Guitart-Masip, Gusalija Behnisch, Constanze I. Seidenbecher, Björn H. Schott

**Affiliations:** 1 Department of Behavioral Neurology, Leibniz Institute for Neurobiology, Brenneckestr. 6, 39118 Magdeburg, Germany; 2 Ageing Research Centre, Karolinska Institute, Stockholm, Sweden; 3 Present Address: Max Planck Institute for Human Development, Center for Lifespan Psychology, Berlin, Germany; 4 Max Planck UCL Centre for Computational Psychiatry and Ageing Research, University College London, London, UK; 5 Center for Behavioral Brain Sciences, Magdeburg, Germany; 6 Department of Psychiatry and Psychotherapy, University Medicine Göttingen, Göttingen, Germany; 7 Department of Neurology, University of Magdeburg, Magdeburg, Germany; 8 German Center for Neurodegenerative Diseases (DZNE), Göttingen, Germany

## Correction to: Journal of Neural Transmission 10.1007/s00702-021-02382-4

The original version of this article unfortunately contained a mistake. Order of the figures (not the figure captions) was interchanged.

The corrected figures and captions (Figs. 1, 2, 3, 4) are given in the following page.

**Fig. 1:**
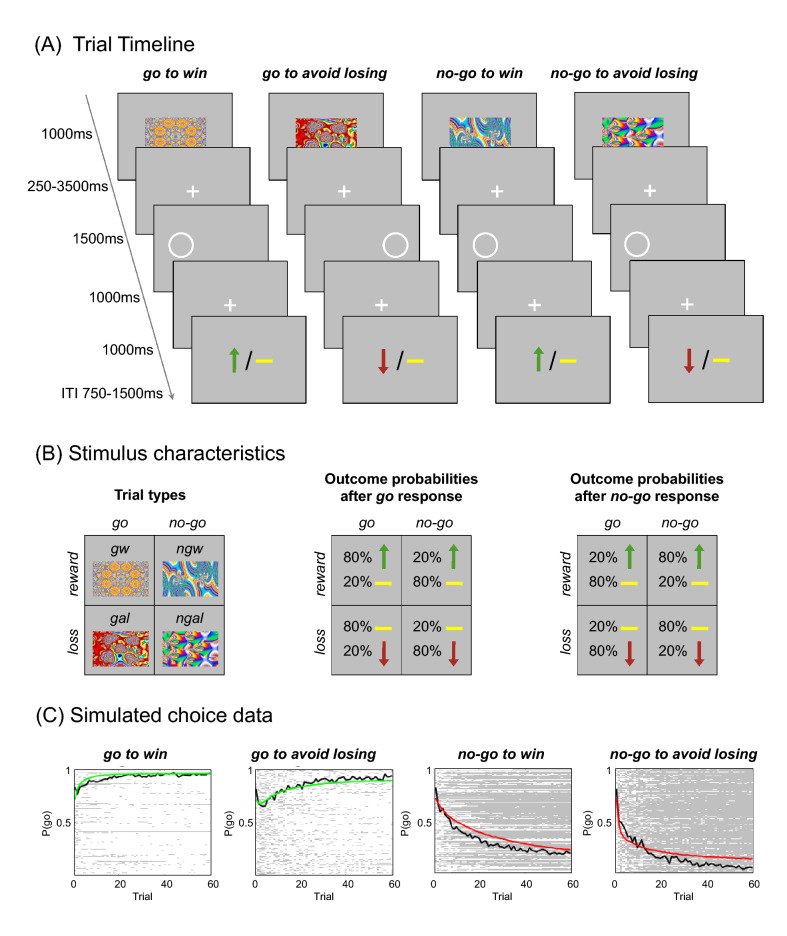
Experimental paradigm and participant performance. **A** Probabilistic monetary *go/no-go* task. Fractal cues indicate the condition—a combination of action (*go* or *no-go*) and valence (*reward* or *punishment*). On *go* trials, subjects press a button for the side of a circle. On *no-go* trials, they withhold a response. Arrows indicate *rewards* (upward) or *punishments* (downward). Horizontal bars symbolize the absence of a *reward* or *punishment*. ITI, intertrial interval. **B** The schematics represent for each condition the nomenclature (left), the possible outcomes and their probabilities after a *go* response (middle), and the possible outcomes and their probability after a *no-go* response (right). **C** Simulated choice data according to the model parameters of the winning model. Colored lines represent the simulated group mean probability of performing a *go* on each trial (green for *go* conditions, where *go* is the correct response; red for *no-go* conditions, where *no-go* is the correct response). Black lines indicate the group mean for participants’ actual *go* responses on each trial. In the plot area, each row represents one participant’s choice behavior for each trial (281 × 60 pixels). A white pixel reflects that a participant chose *go* on that trial; a gray pixel represents *no-go*. Participants made more *go* responses to *win* vs. *avoid losing* cues, reflecting the motivational bias. Overall, they successfully learned whether to make a *go* response or not (proportion of *go* responses increases for *go* cues and decreases for *no-go* cues). Figures (**A**) and (**B**) adapted from Richter et al. (2014)

**Fig. 2:**
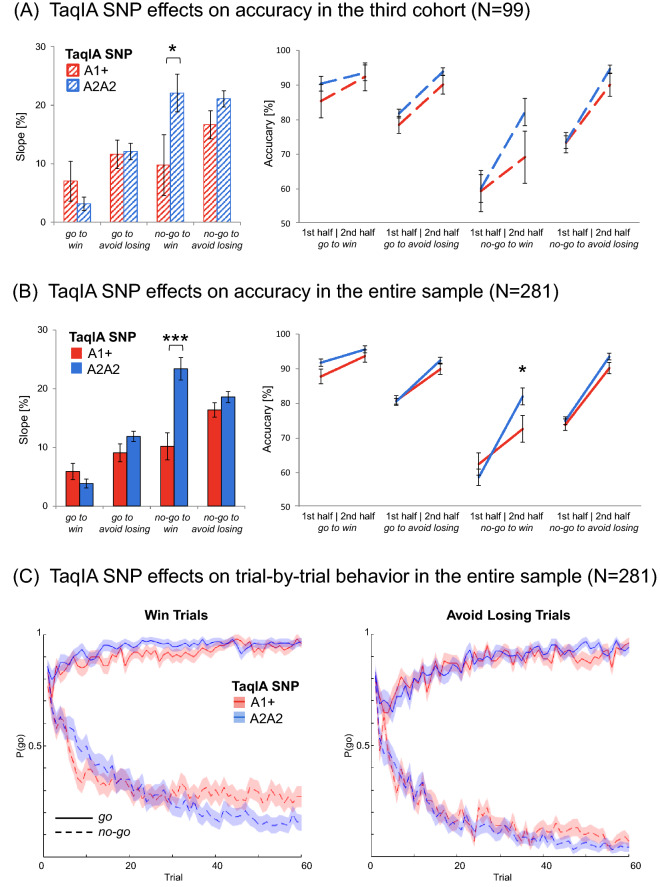
Effects of DRD2/ANKK1 TaqIA genotype on choice performance. **A** and **B** Effects of DRD2/ANKK1 TaqIA genotype on choice performance in the third cohort (*N *= 99) and in the entire sample (*N *= 281). Compared to the A2 homozygotes, A1 carriers showed a diminished learning to withhold an action to receive a reward. Left panels: bar plots show mean differences between correct response rates (± SEM) during second half versus the first half of trials for each condition. This score represents the observed fourfold interaction of *action* × *valence* × *time* × *genotype*. Right panels: line charts show mean values of correct responses (± SEM) in the first and the second half of trials for all four conditions. Post hoc comparisons via *t* tests: *p < 0.05, ***p < 0.001. **C** Trial-by-trial proportions of *go* responses (± SEM) to *go* cues (solid lines) and *no-go* cues (dashed lines) across cue types. *Win* and *avoid losing* condition seperately and colors depict DRD2/ANKK1 TaqIA genotypes. TaqIA A1 carriers showed an enhanced effect of cue valence on *go* responding especially in the *no-go to win* condition with further progress of the experiment (lines are mostly separated). Adapted scripts of Swart et al. (2017) were used to generate figures

**Fig. 3:**
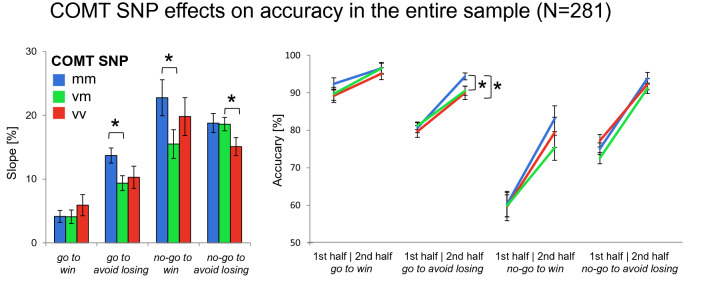
Effects of COMT genotype on choice performance in the entire sample. Left panels: bar plots show mean differences between correct response rates (± SEM) during second half versus the first half of trials for each condition. This score represents the observed fourfold interaction of *action* × *valence* × *time* × *genotype*. Right panels: line charts show mean values of correct responses (± SEM) in the first and the second half of trials for all four conditions. Met homozygotes showed increased learning throughout the experiment in the *no-go to win* and *go avoid losing* condition relative to heterozygotes. Post hoc comparisons via *t* tests: *p < 0.05

**Fig. 4:**
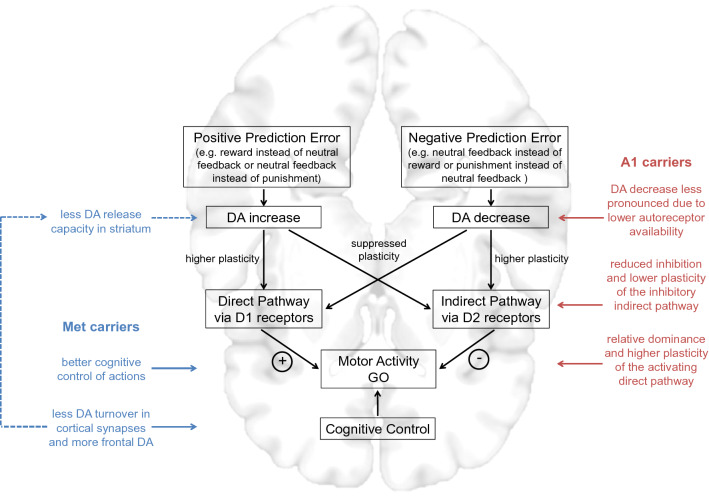
A model of genetically driven contributions to the coupling of action and valence during learning. DA neurons signal positive reward prediction errors by phasic bursts and negative prediction errors by dips below baseline firing rate. While the first reinforces the direct pathway via activation of D1 receptors and thereby facilitates the future generation of *go* choices, the second reinforces the indirect pathway via reduced activation of D2 receptors and thus facilitates the future generation of *no-go* choices in comparable situations. A1 carriers would be assumed to have reduced D2 receptor-binding capacity decreasing autoinhibition of dopaminergic signaling after negative prediction errors in the indirect pathway and a shift to a more action-oriented behavioral pattern mediated by the direct pathway. COMT Val108/158Met Met carriers would be assumed to have higher frontal DA availability facilitating working memory and attentional processes. Moreover, indirect downstream effects on striatal DA regulation may add on improving performance under Pavlovian conflict in Met compared to Val homozygotes. The MNI template brain from MRIcroGL (“mni152”) was used in this illustration. Figure adapted from Richter et al. (2014)

The original article has been corrected.

