# Supplementary material for: Motivational learning biases are differentially modulated by genetic determinants of striatal and prefrontal dopamine function
Source: J Neural Transm (Vienna). 2021 Jul 24;128(11):1705–20. doi: 10.1007/s00702-021-02382-4 (PMC8536632; doi:10.1007/s00702-021-02382-4)
Supplement: Supplementary file 1 — Supplementary file1 (DOCX 902 KB) [file 702_2021_2382_MOESM1_ESM.docx]

**– Supplementary Online Material –**

**Motivational learning biases are differentially modulated by genetic determinants of striatal and prefrontal dopamine function**

Anni Richter^1,#^, Lieke de Boer^2,3^, Marc Guitart-Masip^2,4^, Gusalija Behnisch^1^, Constanze I. Seidenbecher^1,5^, & Björn H. Schott^1,5,6,7,8^

^1^Department of Behavioral Neurology, Leibniz Institute for Neurobiology, Magdeburg, Germany

^2^Ageing Research Centre, Karolinska Institute, Stockholm, Sweden

^3^present address: Max Planck Institute for Human Development, Center for Lifespan Psychology, Berlin, Germany

^4^Max Planck UCL Centre for Computational Psychiatry and Ageing Research, University College London, London, United Kingdom

^5^Center for Behavioral Brain Sciences, Magdeburg, Germany

^6^Department of Psychiatry and Psychotherapy, University Medicine Göttingen, Göttingen, Germany

^7^Department of Neurology, University of Magdeburg, Magdeburg, Germany

^8^German Center for Neurodegenerative Diseases (DZNE), Göttingen, Germany

**Supplementary Introduction**

**
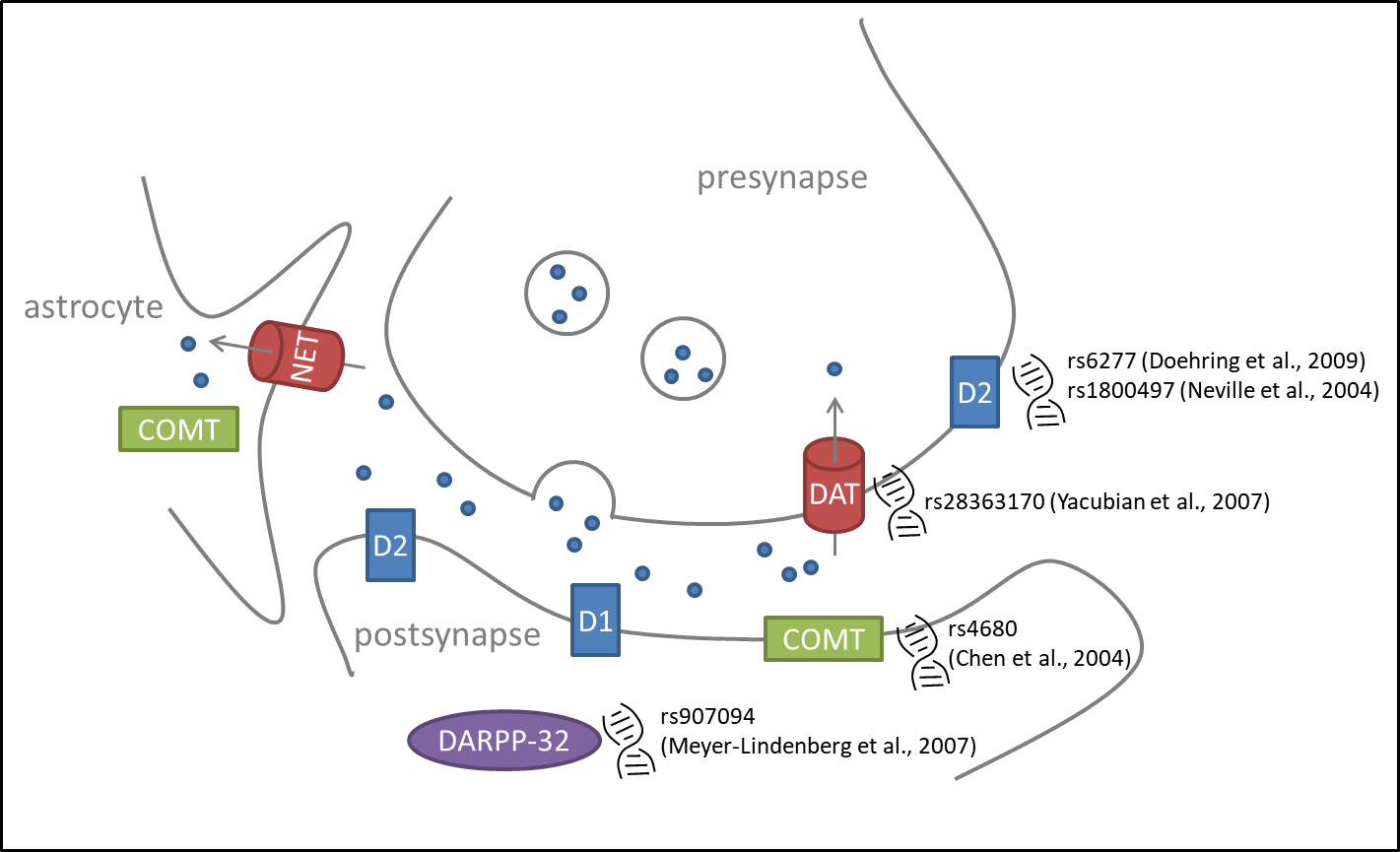
**

**Fig. S1. Simplified schematic illustration of a dopaminergic synapse including commonly investigated polymorphisms that have been associated with alterations in dopaminergic gene products.** COMT: catechol-O-methyltransferase, rs4680 (Chen *et al.*, 2004); DAT: dopamine transporter, rs28363170 (Yacubian *et al.*, 2007); NET: norepinephrine transporter (Kaenmaki *et al.*, 2010); DARPP-32: dopamine- and cAMP-regulated phosphoprotein of molecular weight 32 kDa, rs907094 (Meyer-Lindenberg *et al.*, 2007); dopamine D1 and D2 receptors, rs6277 (Doehring *et al.*, 2009), rs1800497 (Neville *et al.*, 2004). See supplementary discussion for details regarding the polymorphisms.

**Supplementary Methods**

**Supplementary Table S1. Demographic description of the samples**

|  | Cohort 1 | Cohort 2 | Cohort 3 |
| --- | --- | --- | --- |
| Gender (N Women/Men) | 43/44 | 48/47 | 55/44 |
| Age in years (M +/- SD) | 24.6 +/- 3.1 | 24.6 +/- 2.8 | 25.2 +/- 2.6 |
| Age range in years | 19 - 36 | 20 - 33 | 20 - 34 |

N = number, M = mean, SD = standard deviation.

**Trial-by-trial behavioral analysis of *go* responses**

To account for both between- and within-subjects variability, individual *go/no-go* responses (1 = *go*, 0 = *no-go*), were analyzed with logistic mixed-level models using the lme4 package in R 3.5.0 (Bates D, 2014; Team, 2015). The model included all main effects and interactions of the factors action (1 = required *go*, 0 = required *no-go*), valence (1 = *win*, 0 = *avoid losing*) and time (0 = first half, 1 = second half) and a full random effects structure (Barr, 2013; Barr *et al.*, 2013; Swart *et al.*, 2017). A model containing all interactions of the genotypes with the task conditions did not converge. Therefore, we reduced the model according to our ANCOVA results and tested only the effects of each genotype on the time-dependent valence effect on individual *go/no-go* responses (*genotype x valence x time*).

**Supplementary Results**

**Task effects**

In our third independent cohort, we replicated previous results of studies using the same task (Guitart-Masip *et al.*, 2012; Cavanagh *et al.*, 2013; Chowdhury *et al.*, 2013; Guitart-Masip *et al.*, 2014; Richter *et al.*, 2014; de Berker *et al.*, 2016; Swart *et al.*, 2017; Swart *et al.*, 2018; de Boer *et al.*, 2019; Dorfman & Gershman, 2019; Betts *et al.*, 2020; Kuhnel *et al.*, 2020; van Nuland *et al.*, 2020). For an overview of the statistics, see Supplementary Table S2. We observed the main effects of *action* (*F*_1,98_ = 64.55, *p*< .001), reflecting the overall *action* bias, and of *time* (*F*_1,98_ = 242.48, *p* < .001), reflecting learning from the first to the second half of the experiment. Moreover, we observed the known interaction of *action* x *valence* (*F*_1,98_ = 30.32, *p* < .001) that was driven by the comparatively low performance in the *no-go to win* condition. Furthermore, we observed a main effect of valence (*F*_1,98_ = 6.16, *p* = .015), and interactions of time with valence (*F*_1,98_ = 6.52, *p* = .012) and time with action (*F*_1,98_ = 40.23, *p* < .001).

**Supplementary Table S2.** An overview of the statistics in the third cohort

| effect | df | *F* | *p* |
| --- | --- | --- | --- |
| time | 1.98 | 242.84 | **<.001** |
| action | 1.98 | 64.55 | **<.001** |
| valence | 1.98 | 6.16 | **.015** |
| time * action | 1.98 | 46.23 | **<.001** |
| time * valence | 1.98 | 6.52 | **.012** |
| action * valence | 1.98 | 30.32 | **<.001** |
| time * action * valence | 1.98 | 2.80 | .097 |

Time (1st/2nd half), action (go/no-go), and valence (win/avoid losing) as within-subject factors. Boldface type: *p*< .05.

**Supplementary Table S3.** Genotyped polymorphisms in the whole cohort.

| **Gene / SNP** | **rs number** | **Genotype frequencies of the sample** | **HW equilibrium** | **Frequency of the Alternative Allele sample/ALFA** |
| --- | --- | --- | --- | --- |
| ***DRD2/ANKK1 TaqIA*** | rs1800497 | 10/89/182 a1a1/a1a2/a2a2 | *χ*^2^=0.05. *p*=0.828 | 19.4/19.1 |
| ***COMT Val108/158Met*** | rs4680 | 83/128/70 mm/vm/vv | *χ*^2^=2.13. *p*=0.145 | 52.3/50.8 |
| ***BDNF Val66Met*** | rs6265 | 13/82/186 mm/vm/vv | *χ*^2^=1.34. *p*=0.247 | 19.2/19.4 |
| ***RASGRF1*** | rs8027411 | 79/128/74 tt/gt/gg | *χ*^2^=1.02. *p*=0.314 | 50.9/51.2 |
| ***KIBRA*** | rs17070145 | 122/133/26 cc/ct/tt | *χ*^2^=1.44. *p*=0.229 | 32.9/32.4 |
| ***CACNA1C*** | rs1006737 | 35/127/119 aa/ag/gg | *χ*^2^=0.02. *p*=0.901 | 35.1/32.8 |
| ***Neurocan*** | rs1064395 | 6/76/199 aa/ag/gg | *χ*^2^=0.16. *p*=0.688 | 15.7/16.9 |
| ***IL-6*** | rs1800795 | 93/132/56 gg/cg/cc | *χ*^2^=1.64. *p*=0.200 | 56.6/56.0 |

SNP: single nucleotide polymorphism. Note that only the DRD2/ANKK1 TaqIA and the COMT Val108/158Met SNP were subject of the current study investigating dopaminergic polymorphisms. NCBI rs numbers, genotype frequencies of the sample, Hardy-Weinberg (HW) equilibrium and a comparison of the alternative allele frequencies of the current sample and a European population derived from the NCBI ALFA project (Release Version: 20201027095038; (Phan, 2020)) are shown for each SNP.

**Supplementary Table S4.** An overview of the ANCOVA results in the whole cohort.

| effect | df | *F* | *p* |
| --- | --- | --- | --- |
| time | 1, 271 | 9.43 | **.002** |
| time * cohort2 | 1, 271 | 1.80 | .181 |
| time * cohort3 | 1, 271 | 2.65 | .105 |
| time * gender | 1, 271 | 0.18 | .676 |
| time * age | 1, 271 | 0.60 | .439 |
| time * TAQ | 1, 271 | 11.08 | **.001** |
| time * COMT | 2, 271 | 2.51 | .083 |
| time * TAQ * COMT | 2, 271 | 2.39 | .093 |
| action | 1, 271 | 0.31 | .582 |
| action * cohort2 | 1, 271 | 1.02 | .313 |
| action * cohort3 | 1, 271 | 1.01 | .316 |
| action * gender | 1, 271 | 0.02 | .884 |
| action * age | 1, 271 | 1.06 | .304 |
| action * TAQ | 1, 271 | 0.01 | .937 |
| action * COMT | 2, 271 | 0.62 | .539 |
| action * TAQ * COMT | 2, 271 | 0.14 | .872 |
| valence | 1, 271 | 0.01 | .922 |
| valence * cohort2 | 1, 271 | 0.07 | .793 |
| valence * cohort3 | 1, 271 | 0.27 | .605 |
| valence * gender | 1, 271 | 0.55 | .459 |
| valence * age | 1, 271 | 0.58 | .448 |
| valence * TAQ | 1, 271 | 0.00 | .962 |
| valence * COMT | 2, 271 | 0.47 | .627 |
| valence * TAQ * COMT | 2, 271 | 2.20 | .112 |
| time * action | 1, 271 | 0.06 | .814 |
| time * action * cohort2 | 1, 271 | 1.16 | .282 |
| time * action * cohort3 | 1, 271 | 0.31 | .581 |
| time * action * gender | 1, 271 | 1.67 | .197 |
| time * action * age | 1, 271 | 0.10 | .754 |
| time * action * TAQ | 1, 271 | 11.94 | **.001** |
| time * action * COMT | 2, 271 | 0.22 | .801 |
| time * action * TAQ * COMT | 2, 271 | 0.93 | .398 |
| time * valence | 1, 271 | 0.64 | .425 |
| time * valence * cohort2 | 1, 271 | 0.02 | .877 |
| time * valence * cohort3 | 1, 271 | 1.18 | .279 |
| time * valence * gender | 1, 271 | 0.32 | .573 |
| time * valence * age | 1, 271 | 0.19 | .660 |
| time * valence * TAQ | 1, 271 | 3.07 | .081 |
| time * valence * COMT | 2, 271 | 0.87 | .419 |
| time * valence * TAQ * COMT | 2, 271 | 0.33 | .719 |

Continued on next page

**Supplementary Table S4** (continued)

| effect | df | *F* | *p* |
| --- | --- | --- | --- |
| action * valence | 1, 271 | 0.88 | .348 |
| action * valence * cohort2 | 1, 271 | 0.44 | .508 |
| action * valence * cohort3 | 1, 271 | 1.21 | .273 |
| action * valence * gender | 1, 271 | 0.44 | .508 |
| action * valence * age | 1, 271 | 0.29 | .593 |
| action * valence * TAQ | 1, 271 | 0.56 | .457 |
| action * valence * COMT | 2, 271 | 0.11 | .894 |
| action * valence * TAQ * COMT | 2, 271 | 0.48 | .622 |
| time * action * valence | 1, 271 | 2.77 | .097 |
| time * action * valence * cohort2 | 1, 271 | 2.47 | .117 |
| time * action * valence * cohort3 | 1, 271 | 0.67 | .416 |
| time * action * valence * gender | 1, 271 | 0.04 | .843 |
| time * action * valence * age | 1, 271 | 2.79 | .096 |
| time * action * valence * TAQ | 1, 271 | 11.18 | **.001** |
| time * action * valence * COMT | 2, 271 | 2.96 | .053 |
| time * action * valence * TAQ * COMT | 2, 271 | 0.01 | .987 |
| cohort2 | 1, 271 | 6.77 | **.010** |
| cohort3 | 1, 271 | 2.60 | .108 |
| gender | 1, 271 | 3.55 | .061 |
| age | 1, 271 | 6.67 | **.010** |
| TAQ | 1, 271 | 0.97 | .327 |
| COMT | 2, 271 | 2.53 | .082 |
| TAQ * COMT | 2, 271 | 2.03 | .133 |

Time (1st/2nd half), action (go/no-go), and valence (win/avoid losing) as within-subject factors, genotypes as between-subject factors, cohorts (three cohorts represented in two dichotomous dummy-coded variables for cohort 2 and 3), age and gender as covariates. Boldface type: *p*< .05.

**Supplementary Table S5.** An overview of the *post hoc* comparisons in the whole cohort

| variable | TaqIA | N | Mean | SD | *t* | df | *p* | Cohen's *d* |
| --- | --- | --- | --- | --- | --- | --- | --- | --- |
| all conditions 1st half | A1 carriers | 99 | 76.18 | 14.95 | -0.15 | 173.28 | 0.879 |  |
|  | A2 homozygotes | 182 | 76.45 | 12.51 |  |  |  |  |
| all conditions 2nd half | A1 carriers | 99 | 86.55 | 16.95 | -2.21 | 158.90 | **0.028** | 0.35 |
|  | A2 homozygotes | 182 | 90.85 | 12.69 |  |  |  |  |
| go conditions 1st half | A1 carriers | 99 | 84.28 | 14.30 | -1.18 | 146.28 | 0.241 |  |
|  | A2 homozygotes | 182 | 86.16 | 9.52 |  |  |  |  |
| go conditions 2nd half | A1 carriers | 99 | 91.75 | 12.72 | -1.51 | 169.00 | 0.133 |  |
|  | A2 homozygotes | 182 | 94.00 | 10.32 |  |  |  |  |
| nogo conditions 1st half | A1 carriers | 99 | 68.08 | 20.57 | 0.55 | 279.00 | 0.583 |  |
|  | A2 homozygotes | 182 | 66.73 | 19.18 |  |  |  |  |
| nogo conditions 2nd half | A1 carriers | 99 | 81.35 | 24.06 | -2.28 | 164.61 | **0.024** | 0.29 |
|  | A2 homozygotes | 182 | 87.70 | 18.88 |  |  |  |  |
| go to win condition 1st half | A1 carriers | 99 | 87.78 | 21.18 | -1.68 | 147.19 | 0.094 |  |
|  | A2 homozygotes | 182 | 91.78 | 14.23 |  |  |  |  |
| go to avoid losing condition 1st half | A1 carriers | 99 | 80.77 | 14.13 | 0.14 | 163.80 | 0.891 |  |
|  | A2 homozygotes | 182 | 80.55 | 11.02 |  |  |  |  |
| no-go to win condition 1st half | A1 carriers | 99 | 62.39 | 31.99 | 0.96 | 279.00 | 0.34 |  |
|  | A2 homozygotes | 182 | 58.57 | 31.99 |  |  |  |  |
| no-go to avoid losing condition 1st half | A1 carriers | 99 | 73.77 | 16.48 | -0.58 | 279.00 | 0.564 |  |
|  | A2 homozygotes | 182 | 74.89 | 15.00 |  |  |  |  |
| go to win condition 2nd half | A1 carriers | 99 | 93.67 | 17.63 | -1.02 | 279.00 | 0.309 |  |
|  | A2 homozygotes | 182 | 95.60 | 13.70 |  |  |  |  |
| go to avoid losing condition 2nd half | A1 carriers | 99 | 89.83 | 14.93 | -1.49 | 162.35 | 0.139 |  |
|  | A2 homozygotes | 182 | 92.40 | 11.51 |  |  |  |  |
| no-go to win condition 2nd half | A1 carriers | 99 | 72.56 | 38.38 | -2.06 | 175.98 | **0.041** | 0.26 |
|  | A2 homozygotes | 182 | 81.94 | 32.74 |  |  |  |  |
| no-go to avoid losing condition 2nd half | A1 carriers | 99 | 90.13 | 16.16 | -1.76 | 168.82 | 0.081 |  |
|  | A2 homozygotes | 182 | 93.46 | 13.09 |  |  |  |  |
| slope all conditions | A1 carriers | 99 | 10.37 | 7.79 | -3.72 | 279.00 | **<0.001** | 0.47 |
|  | A2 homozygotes | 182 | 14.40 | 9.14 |  |  |  |  |
| slope go conditions | A1 carriers | 99 | 7.47 | 9.83 | -0.32 | 279.00 | 0.748 |  |
|  | A2 homozygotes | 182 | 7.84 | 8.59 |  |  |  |  |
| slope nogo conditions | A1 carriers | 99 | 13.27 | 12.81 | -4.56 | 279.00 | **<0.001** | 0.58 |
|  | A2 homozygotes | 182 | 20.97 | 13.88 |  |  |  |  |
| slope go to win condition | A1 carriers | 99 | 5.89 | 13.68 | 1.31 | 158.91 | 0.191 |  |
|  | A2 homozygotes | 182 | 3.83 | 10.25 |  |  |  |  |
| slope go to avoid losing condition | A1 carriers | 99 | 9.06 | 15.08 | -1.72 | 279.00 | 0.087 |  |
|  | A2 homozygotes | 182 | 11.85 | 11.77 |  |  |  |  |
| slope no-go to win condition | A1 carriers | 99 | 10.17 | 22.94 | -4.41 | 221.84 | **<0.001** | 0.54 |
|  | A2 homozygotes | 182 | 23.37 | 25.74 |  |  |  |  |
| slope no-go to avoid losing condition | A1 carriers | 99 | 16.36 | 12.14 | -1.42 | 279.00 | 0.158 |  |
|  | A2 homozygotes | 182 | 18.57 | 12.65 |  |  |  |  |

Boldface type: *p*< .05.

**Trial-by-trial behavioral analysis of *go* responses**

A summary of the trial-by-trial behavioral analysis is presented in Figure S2. Subjects successfully learned the task and were able to adjust *go* responses to the required action as evidenced by a significantly positive effect of *action* (*z* = 31.16, *p* < .001). A significantly negative effect of *time* (*z* = -18.58, *p* < .001) indicated that they started the experiment with a *go* bias, but could improve over time as evident in a significant *action* x *time* interaction (*z* = 21.03, *p* < .001). As expected, there was a motivational bias in *go* responding revealed by a significant *valence* effect (*z* = 5.44, *p* < .001) akin to the *action* x *valence* interaction for accuracy and hence our main effect of interest. This *valence* effect was stronger for *go* cues as evident in a significantly positive *action* x *valence* interaction (*z* = 6.85, *p* < .001).

In line with the ANCOVA results on accuracy, DRD2 TaqIA A1 carriers showed a more pronounced effect of cue valence on *go* responding specifically at the second half of the experiment (Figure 2C), which was reflected by a significantly negative *genotype* x *valence* x *time* interaction (*z* = 2.62, *p* = .009). For the COMT SNP no interaction with the time-dependent valence effect on individual *go/no-go* responses could be observed (*p* = .381; see Figure S3).


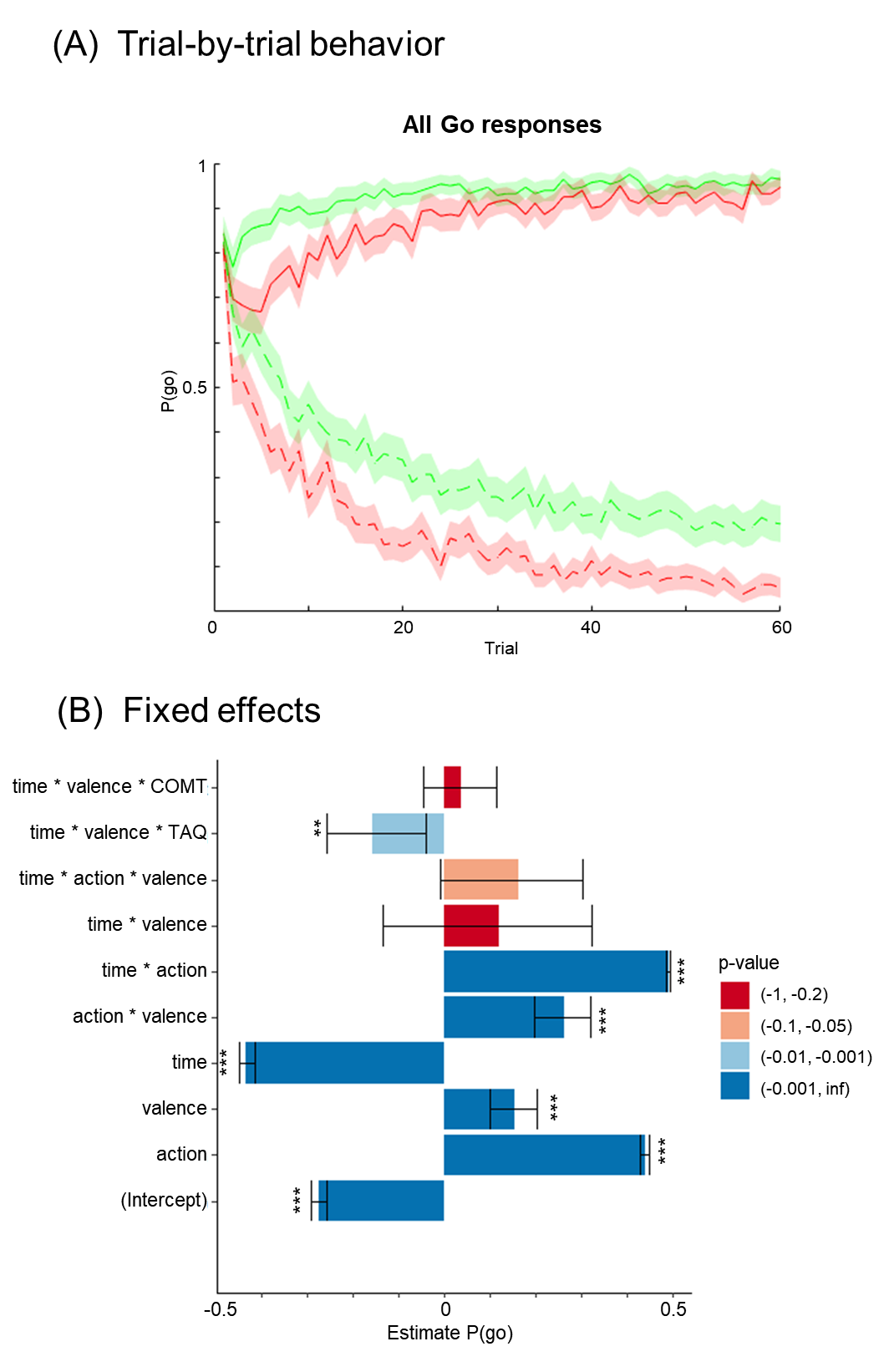


**Fig. S2. Trial-by-trial behavior.** *(A)* Trial-by-trial proportions of *go* responses (±SEM) to *go* cues (solid lines) and *no-go* cues (dashed lines) across cue types. Participants made more *go* responses to *win* vs. *avoid losing* cues (i.e. green lines are above red lines), reflecting the motivational bias. Overall, they successfully learned whether to make a *go* response or not (proportion of *go* responses increases for *go* cues and decreases for *no-go* cues). *(B)* Logistic mixed model estimates of the probability of *go* responses. Fixed effect estimates and 95% confidence interval (CI) are plotted on probability scale. **p* < 0.05, ***p* < 0.01, ****p* < 0.001. Adapted scripts of Swart et al. (Swart *et al.*, 2017) were used to generate figures.


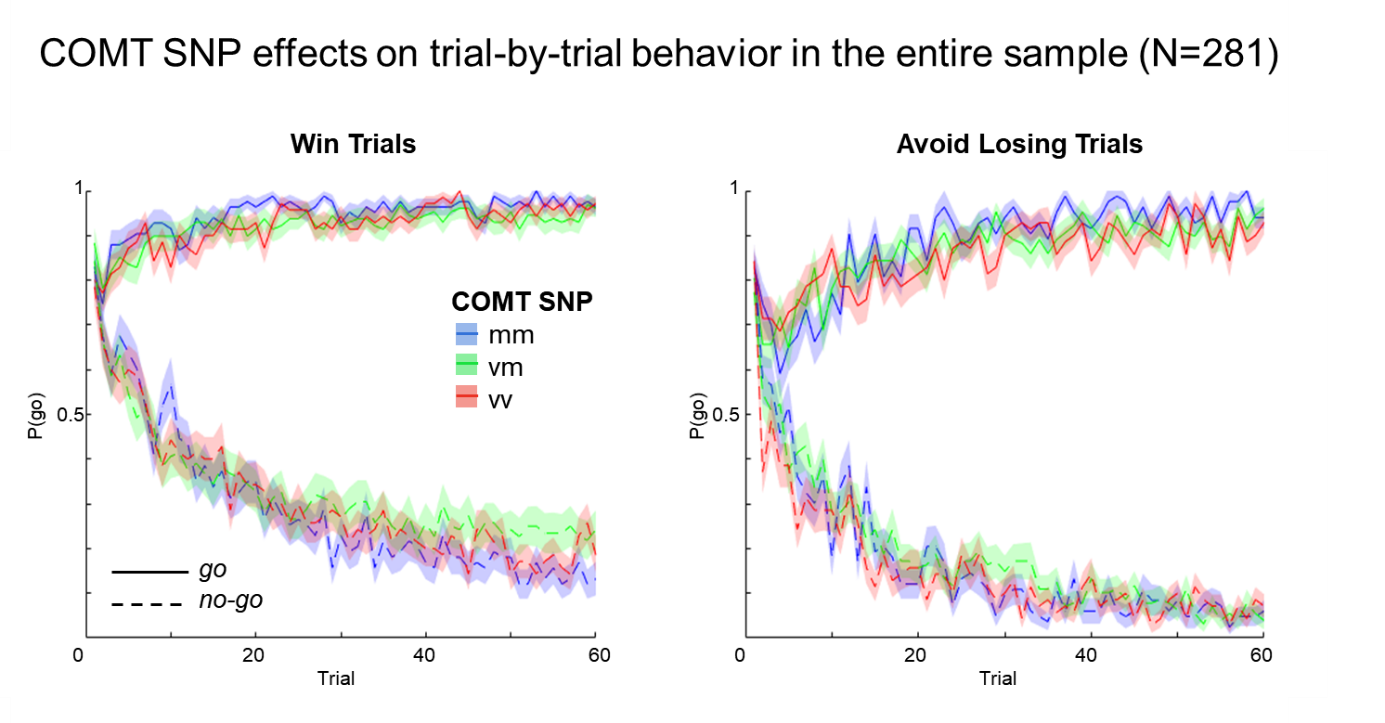


**Fig. S3. Effects of COMT genotype on *go* responses in the entire sample.** Trial-by-trial proportions of go responses (±SEM) to go cues (solid lines) and no-go cues (dashed lines) across cue types. Win and avoid losing condition seperately and colors depict COMT genotypes. Adapted scripts of Swart et al. (Swart *et al.*, 2017) were used to generate figures.

**Analysis of reaction times**

As in our previous study (Richter *et al.*, 2014), the analysis of the reaction times (RTs) in the *go* conditions revealed a *valence* effect (*F*_1,275_ = 32.28, *p* < .001) with faster RTs in the *win* (M +/- SD = 542 +/- 99 ms) compared to the *avoid losing* (M +/- SD = 563 +/- 107 ms) condition (paired *t-*test: *t*_280_ = -5.76, *p* < .001). No effects of *time* or *genotype* were observed (*p* > .221).

**Analysis of false responses**

As a control, we investigated the number of trials where participants responded incorrectly (i.e., left when the target was on the right side of the display or vice versa). False response rates were very low (M +/- SD = 0.5+/-2.0%) and did not differ between genotype groups (TaqIA: Mann-Whitney *U*-test *p* = .403; COMT: Kruskal-Wallis *H*-test *p* = .187), thus ruling out the possibility that genotype effects could be driven by differences in the target detection performance.

**Supplementary Discussion**

**Potential molecular mechanisms of the DRD2/ANKK1 TaqIA polymorphism**

ANKK1, also known as receptor interacting protein 5 (Garrido *et al.*, 2011), codes a potential kinase the function of which is largely unknown. Ankyrin repeat domains are frequent sequential motives that regulate protein-protein interactions and influence the stability, folding and unfolding, as well as the binding properties of proteins (Li *et al.*, 2006). As the *DRD2* and *ANKK1* genes are closely linked (Neville *et al.*, 2004; Ponce *et al.*, 2009), it has been suggested that genetic variations in linkage disequilibrium (LD) with TaqIA might explain the observed relationship between the SNP and alterations of human dopaminergic neurotransmission. Indeed the *DRD2/ANKK1* TaqIA polymorphism is in LD with several polymorphisms on the DRD2 gene of which especially the C957T polymorphism has received considerable attention (Duan *et al.*, 2003; Ritchie & Noble, 2003; Fossella *et al.*, 2006; Doehring *et al.*, 2009; Richter *et al.*, 2017). Until now the number of studies investigating *ANKK1* and *DRD2* polymorphisms together is small and their results are complex. While some postulate the highest risk by the combination of both variants of TaqIA and C957T polymorphism (Morton *et al.*, 2006; Ponce *et al.*, 2008; Yang *et al.*, 2008; Doehring *et al.*, 2009; Hirvonen *et al.*, 2009; Swagell *et al.*, 2012; Voisey *et al.*, 2012), others show that TaqIA effects are carried by the C957T polymorphism (Frank & Hutchison, 2009), and yet others suggest a modulatory effect of the *ANKK1* gene independent of the DRD2 gene (Laakso *et al.*, 2005; Gelernter *et al.*, 2006; Dick *et al.*, 2007; Joutsa *et al.*, 2014). The latter, for example, was confirmed in studies that investigated different variants in the *NCAM1-TTC12-ANKK1-DRD2* region and found the highest associations between addiction disorder and polymorphisms on the *ANKK1* gene (Gelernter *et al.*, 2006; Dick *et al.*, 2007). But also PET studies showing effects of TaqIA but not C957T polymorphism on DA activity affirm this assumption (Laakso *et al.*, 2005; Joutsa *et al.*, 2014). However, one should be careful with associations of *ANKK1* variants, DRD2 expression and a phenotype (Lucht & Rosskopf, 2008). Anyhow current studies point out that *ANKK1* might be involved in a regulatory signaling cascade, that influences the dopaminergic system and especially D2 receptors, what could potentially be modulated by the TaqIA polymorphism (Hoenicka *et al.*, 2010; Garrido *et al.*, 2011; Ponce *et al.*, 2016); for a review, see (Ponce *et al.*, 2009)).

**COMT-dependent clearance of cortical dopamine**

Despite the wide agreement regarding the preferential role for COMT in prefrontal versus striatal dopamine (DA) inactivation, the contributing cellular mechanisms have been subject to considerable debate. Particularly, the question whether COMT can be active in the extracellular space or whether a form of DAT-independent DA uptake is required before COMT-dependent DA inactivation, has yielded conflicting results in previous research. Most of the debate concerns membrane-bound COMT (MB-COMT), the predominant form in neurons and glial cells (Myohanen *et al.*, 2010). In a frequently cited publication, Chen et al. (Chen *et al.*, 2011) reported a series of experiments from which they deduced that neuronal MB-COMT is oriented extracellularly and exhibits extracellular enzymatic activity. The authors could demonstrate the expression of COMT in neurons, but stainings were performed in permeabilized cells and did thus not provide information about intra- versus extracellular localization of epitopes. On the other hand, live staining experiments have shown that COMT epitopes can only be detected after permeabilization of the cell membrane (Ulmanen *et al.*, 1997; Schott *et al.*, 2010).

Even if one was to assume the occasional presence of extracellular COMT catalytic domains, an important question is whether they could be enzymatically active. COMT activity depends on magnesium (Mg^2+^) ions, and it is inhibited by calcium (Ca^2+^) (Sparta & Alexandrova, 2012). Intracellular Mg^2+^ reaches a concentration of 5 to 20 mmol/l, whereas extracellular Mg^2+^ concentrations range from 0.6 to 1.0 mmol/l (Jahnen-Dechent & Ketteler, 2012). Conversely, Ca^2+^ is the predominant divalent cation in the extracellular space (2.2 – 2.6 mmol/l), while its intracellular concentration is low. In their article, Chen et al. (Chen *et al.*, 2011) described that presumably extracellular COMT activity was measured in a medium containing Tris buffer and magnesium chloride, which is poorly comparable with the natural extracellular milieu where the catalytic domain of COMT would most likely be inhibited by the two-to-fourfold higher concentrations of Ca^2+^ compared to Mg^2+^. In summary, evidence for physiologically relevant extracellular COMT activity must be considered questionable. Therefore, a DAT-independent uptake mechanism must contribute to COMT-dependent DA clearance in the PFC. One study suggests that the norepinephrine transporter (NET) likely contributes to this uptake (Kaenmaki *et al.*, 2010), but additional candidate transport proteins may also play a role in DAT-independent DA uptake (for a discussion, see (Schott *et al.*, 2010)).

**Other dopaminergic polymorphisms involved in motivated behavior**

The extent to which different forms and levels of reward and punishments influence our behavior varies interindividually. Polymorphisms are often used to study natural differences in the dopaminergic system of healthy subjects. Therefore, several polymorphisms have been associated to alterations in dopaminergic gene products (e.g. DRD2, COMT, DAT, DARPP-32; see supplementary Figure S1) and motivated behavior.

*DAT1 polymorphism*

The DA transporter (DAT) regulates the reuptake of DA from the synaptic cleft into the presynapse. The gene encoding DAT is located on chromosome 5 (*SLC6A3* or *DAT1*) and contains a polymorphism in the 3′ untranslated region characterized by a variable number of consecutive sequence repeats (variable number of tandem repeats, VNTR; rs28363170). The 9R and 10R alleles are most common, particularly in European populations (Kang *et al.*, 1999). Findings on the influence of polymorphism on *DAT1* gene expression are inconsistent. While some *in vitro* studies suggest that the 10R allele is associated with higher *DAT1* expression (Fuke *et al.*, 2001; Mill *et al.*, 2002; VanNess *et al.*, 2005), others show higher expression in 9R carriers (Michelhaugh *et al.*, 2001; Miller & Madras, 2002) or no association (Greenwood & Kelsoe, 2003). A similar picture emerges in *in vivo* studies, however, the majority of single photon emission computed tomography and positron emission tomography studies performed in healthy subjects show higher DAT density in 9R carriers as opposed to 10R homozygotes (meta-analysis by (Costa *et al.*, 2011); (Shumay *et al.*, 2011; Spencer *et al.*, 2013)). The inconsistent findings make it difficult to reliably infer the influence of the polymorphism on dopaminergic neurotransmission. For a summary presentation and possible interpretation, see the review by Yildirim and Derksen (Yildirim & Derksen, 2015).

Equally inconsistent are the findings on motivated behaviour and the DAT1/VNTR polymorphism in functional magnetic resonance imaging (fMRI) studies. For example, both the 9R allele (Dreher *et al.*, 2009; Forbes *et al.*, 2009; Aarts *et al.*, 2010) and the 10R allele (Wittmann *et al.*, 2013) have been associated with higher activation of reward-associated areas during anticipation or receipt of reward. Hahn et al. (Hahn *et al.*, 2011) postulated that interindividual differences in reward sensitivity might have an influence on the effects, but an interaction with the COMT Val108/158Met polymorphism (Yacubian *et al.*, 2007; Dreher *et al.*, 2009; Nikolova *et al.*, 2011) is also conceivable. For example, Yacubian et al. (Yacubian *et al.*, 2007) found that ventral striatal activation strongly mapped reward magnitude in COMT Met homozygotes carrying the *DAT1* 9R allele carriers and in COMT Val homozygotes who were homozygous for the *DAT1* 10R allele.

*DARPP-32 polymorphism*

DARPP-32 (dopamine- and cAMP-regulated phosphoprotein of molecular weight 32 kDa) is neurally expressed mainly in the regions that receive dopaminergic input from the substantia nigra and ventral tegmental area - for example, in the striatum (Ouimet *et al.*, 1992). DARPP-32 is thought to modulate synaptic plasticity depending on the activity of DA D1 receptors (Calabresi *et al.*, 2000; Lindskog *et al.*, 2006; Stipanovich *et al.*, 2008). DARPP-32 is encoded by the *PPP1R1B* gene (chromosome 17). In a study by Meyer-Lindenberg et al. (Meyer-Lindenberg *et al.*, 2007), who examined post-mortem brain tissue, greater mRNA expression from the *PPP1R1B* gene was detected in homozygous carriers of a common DARPP-32 haplotype variant compared with carriers of a rare variant. At the same time, the authors were able to associate the common variant with reduced putamen volume and stronger structural and functional connectivity between the putamen and PFC in an fMRI study in healthy individuals and with a higher risk of schizophrenia in a clinical study. With regard to motivated behavior, one of the SNPs studied by Meyer-Lindenberg et al. (Meyer-Lindenberg *et al.*, 2007) (rs907094) was further explored by Frank et al. (Frank *et al.*, 2007; Frank *et al.*, 2009): here, T-allele homozygotes associated with stronger mRNA expression in the common haplotype block showed better learning performance of actions in the reward context compared to carriers of the C-allele.

*DRD2 C957T polymorphism*

The C957T SNP (rs6277) is located 12.6 Kb away from the TaqIA polymorphism on exon 7 of the *DRD2* gene (Voisey *et al.*, 2012). Unlike the TaqIA polymorphism, it results in a synonymous base change that does not cause amino acid changes. Nevertheless, functional changes have been observed. Duan et al. (Duan *et al.*, 2003) showed in *in vitro* experiments that the T variant of the SNP alters the predicted structure of the mRNA and decreases mRNA translation and stability. This is in contrast to *in vivo* binding studies (Hirvonen *et al.*, 2004; Hirvonen *et al.*, 2009): while the results of Hirvonen *et al.* regarding the DRD2/ANKK1 TaqIA polymorphism were in agreement with previous studies, they observed an association between the C allele and decreased DRD2 density for the C957T polymorphism, in contrast to Duan et al. (Duan *et al.*, 2003). The authors explained the discrepancies with the *in vitro* data by the *in vivo* complexity of the regulation of DA neurotransmission in the human brain. In addition, combined analysis of both SNPs showed an association between the A1+/C+ haplotype and the lowest striatal DRD2 availability compared with the other variants (Hirvonen *et al.*, 2009).

In terms of neuropsychological traits, like the A1 allele, the C957T C allele is also associated with impaired punishment learning (Frank *et al.*, 2007; Frank *et al.*, 2009) and, together with the A1 allele in the haplotype, with addictive disorders (Morton *et al.*, 2006; Doehring *et al.*, 2009; Swagell *et al.*, 2012; Voisey *et al.*, 2012).

**References**

Aarts, E., Roelofs, A., Franke, B., Rijpkema, M., Fernandez, G., Helmich, R.C. & Cools, R. (2010) Striatal dopamine mediates the interface between motivational and cognitive control in humans: evidence from genetic imaging. *Neuropsychopharmacology : official publication of the American College of Neuropsychopharmacology*, **35**, 1943-1951.

Barr, D.J. (2013) Random effects structure for testing interactions in linear mixed-effects models. *Front Psychol*, **4**, 328.

Barr, D.J., Levy, R., Scheepers, C. & Tily, H.J. (2013) Random effects structure for confirmatory hypothesis testing: Keep it maximal. *J Mem Lang*, **68**.

Bates D, M.M., Bolker B, Walker S. (2014) lme4: Linear mixed-effects models using Eigen and S4.

Betts, M.J., Richter, A., de Boer, L., Tegelbeckers, J., Perosa, V., Baumann, V., Chowdhury, R., Dolan, R.J., Seidenbecher, C., Schott, B.H., Duzel, E., Guitart-Masip, M. & Krauel, K. (2020) Learning in anticipation of reward and punishment: perspectives across the human lifespan. *Neurobiol Aging*, **96**, 49-57.

Calabresi, P., Gubellini, P., Centonze, D., Picconi, B., Bernardi, G., Chergui, K., Svenningsson, P., Fienberg, A.A. & Greengard, P. (2000) Dopamine and cAMP-regulated phosphoprotein 32 kDa controls both striatal long-term depression and long-term potentiation, opposing forms of synaptic plasticity. *The Journal of neuroscience : the official journal of the Society for Neuroscience*, **20**, 8443-8451.

Cavanagh, J.F., Eisenberg, I., Guitart-Masip, M., Huys, Q. & Frank, M.J. (2013) Frontal theta overrides pavlovian learning biases. *The Journal of neuroscience : the official journal of the Society for Neuroscience*, **33**, 8541-8548.

Chen, J., Lipska, B.K., Halim, N., Ma, Q.D., Matsumoto, M., Melhem, S., Kolachana, B.S., Hyde, T.M., Herman, M.M., Apud, J., Egan, M.F., Kleinman, J.E. & Weinberger, D.R. (2004) Functional analysis of genetic variation in catechol-O-methyltransferase (COMT): effects on mRNA, protein, and enzyme activity in postmortem human brain. *Am J Hum Genet*, **75**, 807-821.

Chen, J., Song, J., Yuan, P., Tian, Q., Ji, Y., Ren-Patterson, R., Liu, G., Sei, Y. & Weinberger, D.R. (2011) Orientation and cellular distribution of membrane-bound catechol-O-methyltransferase in cortical neurons: implications for drug development. *J Biol Chem*, **286**, 34752-34760.

Chowdhury, R., Guitart-Masip, M., Lambert, C., Dolan, R.J. & Duzel, E. (2013) Structural integrity of the substantia nigra and subthalamic nucleus predicts flexibility of instrumental learning in older-age individuals. *Neurobiol Aging*, **34**, 2261-2270.

Costa, A., Riedel, M., Muller, U., Moller, H.J. & Ettinger, U. (2011) Relationship between SLC6A3 genotype and striatal dopamine transporter availability: a meta-analysis of human single photon emission computed tomography studies. *Synapse*, **65**, 998-1005.

de Berker, A.O., Tirole, M., Rutledge, R.B., Cross, G.F., Dolan, R.J. & Bestmann, S. (2016) Acute stress selectively impairs learning to act. *Sci Rep*, **6**, 29816.

de Boer, L., Axelsson, J., Chowdhury, R., Riklund, K., Dolan, R.J., Nyberg, L., Backman, L. & Guitart-Masip, M. (2019) Dorsal striatal dopamine D1 receptor availability predicts an instrumental bias in action learning. *Proceedings of the National Academy of Sciences of the United States of America*, **116**, 261-270.

Dick, D.M., Wang, J.C., Plunkett, J., Aliev, F., Hinrichs, A., Bertelsen, S., Budde, J.P., Goldstein, E.L., Kaplan, D., Edenberg, H.J., Nurnberger, J., Jr., Hesselbrock, V., Schuckit, M., Kuperman, S., Tischfield, J., Porjesz, B., Begleiter, H., Bierut, L.J. & Goate, A. (2007) Family-based association analyses of alcohol dependence phenotypes across DRD2 and neighboring gene ANKK1. *Alcoholism, clinical and experimental research*, **31**, 1645-1653.

Doehring, A., Hentig, N., Graff, J., Salamat, S., Schmidt, M., Geisslinger, G., Harder, S. & Lotsch, J. (2009) Genetic variants altering dopamine D2 receptor expression or function modulate the risk of opiate addiction and the dosage requirements of methadone substitution. *Pharmacogenet Genomics*, **19**, 407-414.

Dorfman, H.M. & Gershman, S.J. (2019) Controllability governs the balance between Pavlovian and instrumental action selection. *Nat Commun*, **10**, 5826.

Dreher, J.C., Kohn, P., Kolachana, B., Weinberger, D.R. & Berman, K.F. (2009) Variation in dopamine genes influences responsivity of the human reward system. *Proceedings of the National Academy of Sciences of the United States of America*, **106**, 617-622.

Duan, J., Wainwright, M.S., Comeron, J.M., Saitou, N., Sanders, A.R., Gelernter, J. & Gejman, P.V. (2003) Synonymous mutations in the human dopamine receptor D2 (DRD2) affect mRNA stability and synthesis of the receptor. *Hum Mol Genet*, **12**, 205-216.

Forbes, E.E., Brown, S.M., Kimak, M., Ferrell, R.E., Manuck, S.B. & Hariri, A.R. (2009) Genetic variation in components of dopamine neurotransmission impacts ventral striatal reactivity associated with impulsivity. *Molecular psychiatry*, **14**, 60-70.

Fossella, J., Green, A.E. & Fan, J. (2006) Evaluation of a structural polymorphism in the ankyrin repeat and kinase domain containing 1 (ANKK1) gene and the activation of executive attention networks. *Cognitive, affective & behavioral neuroscience*, **6**, 71-78.

Frank, M.J., Doll, B.B., Oas-Terpstra, J. & Moreno, F. (2009) Prefrontal and striatal dopaminergic genes predict individual differences in exploration and exploitation. *Nature neuroscience*, **12**, 1062-1068.

Frank, M.J. & Hutchison, K. (2009) Genetic contributions to avoidance-based decisions: striatal D2 receptor polymorphisms. *Neuroscience*, **164**, 131-140.

Frank, M.J., Moustafa, A.A., Haughey, H.M., Curran, T. & Hutchison, K.E. (2007) Genetic triple dissociation reveals multiple roles for dopamine in reinforcement learning. *Proceedings of the National Academy of Sciences of the United States of America*, **104**, 16311-16316.

Fuke, S., Suo, S., Takahashi, N., Koike, H., Sasagawa, N. & Ishiura, S. (2001) The VNTR polymorphism of the human dopamine transporter (DAT1) gene affects gene expression. *Pharmacogenomics J*, **1**, 152-156.

Garrido, E., Palomo, T., Ponce, G., Garcia-Consuegra, I., Jimenez-Arriero, M.A. & Hoenicka, J. (2011) The ANKK1 protein associated with addictions has nuclear and cytoplasmic localization and shows a differential response of Ala239Thr to apomorphine. *Neurotox Res*, **20**, 32-39.

Gelernter, J., Yu, Y., Weiss, R., Brady, K., Panhuysen, C., Yang, B.Z., Kranzler, H.R. & Farrer, L. (2006) Haplotype spanning TTC12 and ANKK1, flanked by the DRD2 and NCAM1 loci, is strongly associated to nicotine dependence in two distinct American populations. *Hum Mol Genet*, **15**, 3498-3507.

Greenwood, T.A. & Kelsoe, J.R. (2003) Promoter and intronic variants affect the transcriptional regulation of the human dopamine transporter gene. *Genomics*, **82**, 511-520.

Guitart-Masip, M., Economides, M., Huys, Q.J., Frank, M.J., Chowdhury, R., Duzel, E., Dayan, P. & Dolan, R.J. (2014) Differential, but not opponent, effects of L -DOPA and citalopram on action learning with reward and punishment. *Psychopharmacology*, **231**, 955-966.

Guitart-Masip, M., Huys, Q.J., Fuentemilla, L., Dayan, P., Duzel, E. & Dolan, R.J. (2012) Go and no-go learning in reward and punishment: interactions between affect and effect. *NeuroImage*, **62**, 154-166.

Hahn, T., Heinzel, S., Dresler, T., Plichta, M.M., Renner, T.J., Markulin, F., Jakob, P.M., Lesch, K.P. & Fallgatter, A.J. (2011) Association between reward-related activation in the ventral striatum and trait reward sensitivity is moderated by dopamine transporter genotype. *Human brain mapping*, **32**, 1557-1565.

Hirvonen, M., Laakso, A., Nagren, K., Rinne, J.O., Pohjalainen, T. & Hietala, J. (2004) C957T polymorphism of the dopamine D2 receptor (DRD2) gene affects striatal DRD2 availability in vivo. *Molecular psychiatry*, **9**, 1060-1061.

Hirvonen, M.M., Laakso, A., Nagren, K., Rinne, J.O., Pohjalainen, T. & Hietala, J. (2009) C957T polymorphism of dopamine D2 receptor gene affects striatal DRD2 in vivo availability by changing the receptor affinity. *Synapse*, **63**, 907-912.

Hoenicka, J., Quinones-Lombrana, A., Espana-Serrano, L., Alvira-Botero, X., Kremer, L., Perez-Gonzalez, R., Rodriguez-Jimenez, R., Jimenez-Arriero, M.A., Ponce, G. & Palomo, T. (2010) The ANKK1 gene associated with addictions is expressed in astroglial cells and upregulated by apomorphine. *Biological psychiatry*, **67**, 3-11.

Jahnen-Dechent, W. & Ketteler, M. (2012) Magnesium basics. *Clin Kidney J*, **5**, i3-i14.

Joutsa, J., Hirvonen, M.M., Arponen, E., Hietala, J. & Kaasinen, V. (2014) DRD2-related TaqIA genotype is associated with dopamine release during a gambling task. *J Addict Med*, **8**, 294-295.

Kaenmaki, M., Tammimaki, A., Myohanen, T., Pakarinen, K., Amberg, C., Karayiorgou, M., Gogos, J.A. & Mannisto, P.T. (2010) Quantitative role of COMT in dopamine clearance in the prefrontal cortex of freely moving mice. *J Neurochem*, **114**, 1745-1755.

Kang, A.M., Palmatier, M.A. & Kidd, K.K. (1999) Global variation of a 40-bp VNTR in the 3'-untranslated region of the dopamine transporter gene (SLC6A3). *Biological psychiatry*, **46**, 151-160.

Kuhnel, A., Teckentrup, V., Neuser, M.P., Huys, Q.J.M., Burrasch, C., Walter, M. & Kroemer, N.B. (2020) Stimulation of the vagus nerve reduces learning in a go/no-go reinforcement learning task. *Eur Neuropsychopharmacol*, **35**, 17-29.

Laakso, A., Pohjalainen, T., Bergman, J., Kajander, J., Haaparanta, M., Solin, O., Syvalahti, E. & Hietala, J. (2005) The A1 allele of the human D2 dopamine receptor gene is associated with increased activity of striatal L-amino acid decarboxylase in healthy subjects. *Pharmacogenet Genomics*, **15**, 387-391.

Li, J., Mahajan, A. & Tsai, M.D. (2006) Ankyrin repeat: a unique motif mediating protein-protein interactions. *Biochemistry*, **45**, 15168-15178.

Lindskog, M., Kim, M., Wikstrom, M.A., Blackwell, K.T. & Kotaleski, J.H. (2006) Transient calcium and dopamine increase PKA activity and DARPP-32 phosphorylation. *PLoS Comput Biol*, **2**, e119.

Lucht, M. & Rosskopf, D. (2008) Comment on "Genetically determined differences in learning from errors". *Science*, **321**, 200; author reply 200.

Meyer-Lindenberg, A., Straub, R.E., Lipska, B.K., Verchinski, B.A., Goldberg, T., Callicott, J.H., Egan, M.F., Huffaker, S.S., Mattay, V.S., Kolachana, B., Kleinman, J.E. & Weinberger, D.R. (2007) Genetic evidence implicating DARPP-32 in human frontostriatal structure, function, and cognition. *J Clin Invest*, **117**, 672-682.

Michelhaugh, S.K., Fiskerstrand, C., Lovejoy, E., Bannon, M.J. & Quinn, J.P. (2001) The dopamine transporter gene (SLC6A3) variable number of tandem repeats domain enhances transcription in dopamine neurons. *J Neurochem*, **79**, 1033-1038.

Mill, J., Asherson, P., Browes, C., D'Souza, U. & Craig, I. (2002) Expression of the dopamine transporter gene is regulated by the 3' UTR VNTR: Evidence from brain and lymphocytes using quantitative RT-PCR. *American journal of medical genetics*, **114**, 975-979.

Miller, G.M. & Madras, B.K. (2002) Polymorphisms in the 3'-untranslated region of human and monkey dopamine transporter genes affect reporter gene expression. *Molecular psychiatry*, **7**, 44-55.

Morton, L.M., Wang, S.S., Bergen, A.W., Chatterjee, N., Kvale, P., Welch, R., Yeager, M., Hayes, R.B., Chanock, S.J. & Caporaso, N.E. (2006) DRD2 genetic variation in relation to smoking and obesity in the Prostate, Lung, Colorectal, and Ovarian Cancer Screening Trial. *Pharmacogenet Genomics*, **16**, 901-910.

Myohanen, T.T., Schendzielorz, N. & Mannisto, P.T. (2010) Distribution of catechol-O-methyltransferase (COMT) proteins and enzymatic activities in wild-type and soluble COMT deficient mice. *J Neurochem*, **113**, 1632-1643.

Neville, M.J., Johnstone, E.C. & Walton, R.T. (2004) Identification and characterization of ANKK1: a novel kinase gene closely linked to DRD2 on chromosome band 11q23.1. *Hum Mutat*, **23**, 540-545.

Nikolova, Y.S., Ferrell, R.E., Manuck, S.B. & Hariri, A.R. (2011) Multilocus genetic profile for dopamine signaling predicts ventral striatum reactivity. *Neuropsychopharmacology : official publication of the American College of Neuropsychopharmacology*, **36**, 1940-1947.

Ouimet, C.C., LaMantia, A.S., Goldman-Rakic, P., Rakic, P. & Greengard, P. (1992) Immunocytochemical localization of DARPP-32, a dopamine and cyclic-AMP-regulated phosphoprotein, in the primate brain. *J Comp Neurol*, **323**, 209-218.

Phan, L.J., Y.; Zhang, H.; Qiang, W.; Shekhtman, E.; Shao, D.; Revoe, D.; Villamarin, R. et al. (2020) ALFA: Allele Frequency Aggregator. *National Center for Biotechnology Information, U.S. National Library of Medicine*.

Ponce, G., Hoenicka, J., Jimenez-Arriero, M.A., Rodriguez-Jimenez, R., Aragues, M., Martin-Sune, N., Huertas, E. & Palomo, T. (2008) DRD2 and ANKK1 genotype in alcohol-dependent patients with psychopathic traits: association and interaction study. *Br J Psychiatry*, **193**, 121-125.

Ponce, G., Perez-Gonzalez, R., Aragues, M., Palomo, T., Rodriguez-Jimenez, R., Jimenez-Arriero, M.A. & Hoenicka, J. (2009) The ANKK1 kinase gene and psychiatric disorders. *Neurotox Res*, **16**, 50-59.

Ponce, G., Quinones-Lombrana, A., Martin-Palanco, N.G., Rubio-Solsona, E., Jimenez-Arriero, M.A., Palomo, T. & Hoenicka, J. (2016) The Addiction-Related Gene Ankk1 is Oppositely Regulated by D1R- and D2R-Like Dopamine Receptors. *Neurotox Res*, **29**, 345-350.

Richter, A., Barman, A., Wustenberg, T., Soch, J., Schanze, D., Deibele, A., Behnisch, G., Assmann, A., Klein, M., Zenker, M., Seidenbecher, C. & Schott, B.H. (2017) Behavioral and Neural Manifestations of Reward Memory in Carriers of Low-Expressing versus High-Expressing Genetic Variants of the Dopamine D2 Receptor. *Front Psychol*, **8**, 654.

Richter, A., Guitart-Masip, M., Barman, A., Libeau, C., Behnisch, G., Czerney, S., Schanze, D., Assmann, A., Klein, M., Duzel, E., Zenker, M., Seidenbecher, C.I. & Schott, B.H. (2014) Valenced action/inhibition learning in humans is modulated by a genetic variant linked to dopamine D2 receptor expression. *Front Syst Neurosci*, **8**, 140.

Ritchie, T. & Noble, E.P. (2003) Association of seven polymorphisms of the D2 dopamine receptor gene with brain receptor-binding characteristics. *Neurochemical research*, **28**, 73-82.

Schott, B.H., Frischknecht, R., Debska-Vielhaber, G., John, N., Behnisch, G., Duzel, E., Gundelfinger, E.D. & Seidenbecher, C.I. (2010) Membrane-Bound Catechol-O-Methyl Transferase in Cortical Neurons and Glial Cells is Intracellularly Oriented. *Front Psychiatry*, **1**, 142.

Shumay, E., Chen, J., Fowler, J.S. & Volkow, N.D. (2011) Genotype and ancestry modulate brain's DAT availability in healthy humans. *PLoS One*, **6**, e22754.

Sparta, M. & Alexandrova, A.N. (2012) How metal substitution affects the enzymatic activity of catechol-o-methyltransferase. *PLoS One*, **7**, e47172.

Spencer, T.J., Biederman, J., Faraone, S.V., Madras, B.K., Bonab, A.A., Dougherty, D.D., Batchelder, H., Clarke, A. & Fischman, A.J. (2013) Functional genomics of attention-deficit/hyperactivity disorder (ADHD) risk alleles on dopamine transporter binding in ADHD and healthy control subjects. *Biological psychiatry*, **74**, 84-89.

Stipanovich, A., Valjent, E., Matamales, M., Nishi, A., Ahn, J.H., Maroteaux, M., Bertran-Gonzalez, J., Brami-Cherrier, K., Enslen, H., Corbille, A.G., Filhol, O., Nairn, A.C., Greengard, P., Herve, D. & Girault, J.A. (2008) A phosphatase cascade by which rewarding stimuli control nucleosomal response. *Nature*, **453**, 879-884.

Swagell, C.D., Lawford, B.R., Hughes, I.P., Voisey, J., Feeney, G.F., van Daal, A., Connor, J.P., Noble, E.P., Morris, C.P. & Young, R.M. (2012) DRD2 C957T and TaqIA genotyping reveals gender effects and unique low-risk and high-risk genotypes in alcohol dependence. *Alcohol Alcohol*, **47**, 397-403.

Swart, J.C., Frank, M.J., Maatta, J.I., Jensen, O., Cools, R. & den Ouden, H.E.M. (2018) Frontal network dynamics reflect neurocomputational mechanisms for reducing maladaptive biases in motivated action. *PLoS Biol*, **16**, e2005979.

Swart, J.C., Frobose, M.I., Cook, J.L., Geurts, D.E., Frank, M.J., Cools, R. & den Ouden, H.E. (2017) Catecholaminergic challenge uncovers distinct Pavlovian and instrumental mechanisms of motivated (in)action. *Elife*, **6**.

Team, R.D.C. (2015) R: a language and environment for Statistical Computing. Vienna, Austria: R

Foundation for Statistical Computing.

Ulmanen, I., Peranen, J., Tenhunen, J., Tilgmann, C., Karhunen, T., Panula, P., Bernasconi, L., Aubry, J.P. & Lundstrom, K. (1997) Expression and intracellular localization of catechol O-methyltransferase in transfected mammalian cells. *Eur J Biochem*, **243**, 452-459.

van Nuland, A.J., Helmich, R.C., Dirkx, M.F., Zach, H., Toni, I., Cools, R. & den Ouden, H.E.M. (2020) Effects of dopamine on reinforcement learning in Parkinson's disease depend on motor phenotype. *Brain : a journal of neurology*, **143**, 3422-3434.

VanNess, S.H., Owens, M.J. & Kilts, C.D. (2005) The variable number of tandem repeats element in DAT1 regulates in vitro dopamine transporter density. *BMC Genet*, **6**, 55.

Voisey, J., Swagell, C.D., Hughes, I.P., van Daal, A., Noble, E.P., Lawford, B.R., Young, R.M. & Morris, C.P. (2012) A DRD2 and ANKK1 haplotype is associated with nicotine dependence. *Psychiatry Res*, **196**, 285-289.

Wittmann, B.C., Tan, G.C., Lisman, J.E., Dolan, R.J. & Duzel, E. (2013) Reprint of: DAT genotype modulates striatal processing and long-term memory for items associated with reward and punishment. *Neuropsychologia*, **51**, 2469-2477.

Yacubian, J., Sommer, T., Schroeder, K., Glascher, J., Braus, D.F. & Buchel, C. (2007) Subregions of the ventral striatum show preferential coding of reward magnitude and probability. *NeuroImage*, **38**, 557-563.

Yang, B.Z., Kranzler, H.R., Zhao, H., Gruen, J.R., Luo, X. & Gelernter, J. (2008) Haplotypic variants in DRD2, ANKK1, TTC12, and NCAM1 are associated with comorbid alcohol and drug dependence. *Alcoholism, clinical and experimental research*, **32**, 2117-2127.

Yildirim, B.O. & Derksen, J.J. (2015) Mesocorticolimbic dopamine functioning in primary psychopathy: A source of within-group heterogeneity. *Psychiatry Res*, **229**, 633-677.
